# Author Correction: Silica accelerates the selective hydrogenation of CO_2_ to methanol on cobalt catalysts

**DOI:** 10.1038/s41467-020-15385-8

**Published:** 2020-04-01

**Authors:** Lingxiang Wang, Erjia Guan, Yeqing Wang, Liang Wang, Zhongmiao Gong, Yi Cui, Xiangju Meng, Bruce C. Gates, Feng-Shou Xiao

**Affiliations:** 1 Key Lab of Biomass Chemical Engineering of Ministry of Education, College of Chemical and Biological Engineering, Zhejiang University, 310027 Hangzhou, China; 2 Key Laboratory of Applied Chemistry of Zhejiang Province, Department of Chemistry, Zhejiang University, 310028 Hangzhou, China; 3 Department of Chemical Engineering, University of California, Davis, CA 95616 USA; 4 Zhejiang Provincial Key Laboratory of Advanced Chemical Engineering Manufacture Technology, Zhejiang University, 310027 Hangzhou, China; 5 Vacuum Interconnected Nanotech Workstation (Nano-X), Suzhou Institute of Nano-Tech and Nano-Bionics (SINANO), Chinese Academy of Sciences (CAS), 215123 Suzhou, China; 6 Beijing Advanced Innovation Center for Soft Matter Science and Engineering, Beijing University of Chemical Technology, 100029 Beijing, China

**Keywords:** Heterogeneous catalysis, Sustainability, Chemical engineering

Correction to: *Nature Communications* 10.1038/s41467-020-14817-9, published online 25 February 2020.

The original version of this Article contained incorrect calculation of methanol productivity according to the experimental data (all the experimental data of CO_2_ conversion and methanol selectivity are correct). The calculated data are incorrectly included in Fig. 2b, Supplementary Fig. 36, and Supplementary Table 3. These data have been corrected in both the PDF and HTML versions of the Article.

