# Supplementary material for: Silica accelerates the selective hydrogenation of CO2 to methanol on cobalt catalysts
Source: Nat Commun. 2020 Feb 25;11:1033. doi: 10.1038/s41467-020-14817-9 (PMC7042257; doi:10.1038/s41467-020-14817-9)
Supplement: Supplementary file 1 — Supplementary Information [file 41467_2020_14817_MOESM1_ESM.pdf]

Silica accelerates the selective hydrogenation of CO_2 to methanol on cobalt catalysts

Wang et al.

Table of Contents

Supplementary Figure 1. XRD patterns of (a) Co_3O_4@Si_x and (b) Co@Si_x catalysts.
Supplementary Figure 2. (a) Dependences of the CO_2 conversion, product selectivity, and (b) carbon balance on temperature over CoO_x catalyst.
Supplementary Figure 3. (a) Dependences of the CO_2 conversion, product selectivity, and (b) carbon balance on temperature over Co@$Si_{0.52}$ catalyst.
Supplementary Figure 4. (a) Dependences of the CO_2 conversion, product selectivity, and (b) carbon balance on temperature over Co@$Si_{0.95}$ catalyst.
Supplementary Figure 5. (a) Dependences of the CO_2 conversion, product selectivity, and (b) carbon balance on temperature over Co@$Si_{1.48}$ catalyst.
Supplementary Figure 6. (a) Dependences of the CO_2 conversion, product selectivity, and (b) carbon balance on temperature over Co@$Si_{1.87}$ catalyst.
Supplementary Figure 7. Data characterizing the influences of reaction pressure, H_2/CO_2 ratio, and velocity to the performances of Co@$Si_{0.95}$ catalyst in CO_2 hydrogenation.
Supplementary Figure 8. Data characterizing the CO_2 conversions and CH_4 selectivity on (a) reaction temperature, (b) pressure, (c) H_2/CO_2 ratio, and (d) velocity over Co/SiO_2 catalyst.
Supplementary Figure 9. XRD patterns of as-synthesized Co_3O_4/SiO_2 and Co/SiO_2.
Supplementary Figure 10. (a) Dependences of the CO_2 conversion, products selectivity, and (b) carbon balance on temperature over Co/SiO_2 catalyst.
Supplementary Figure 11. XRD patterns of CuO/ZnO/Al_2O_3 and Cu/ZnO/Al_2O_3 catalysts.
Supplementary Figure 12. (a) Dependences of the CO_2 conversion, products selectivity, and (b) carbon balance on temperature over Cu/ZnO/Al_2O_3 catalyst.
Supplementary Figure 13. Data showing the durability of Cu/ZnO/Al_2O_3 catalyst in CO_2 hydrogenation.
Supplementary Figure 14. (a, b) TEM images, (c) Co nanoparticle size distribution, and (d) HRTEM image of CoO_x.
Supplementary Figure 15. (a, b) TEM images, (c) Co nanoparticle size distribution, and (d) HRTEM image of Co@$Si_{0.52}$.
Supplementary Figure 16. (a, b) TEM images, (c) Co nanoparticle size distribution, and (d) HRTEM image of Co@$Si_{1.48}$.
Supplementary Figure 17. (a, b) TEM images, (c) Co nanoparticle size distribution, and (d) HRTEM image of Co@$Si_{1.87}$.
Supplementary Figure 18. (a, b) TEM images, (c) Co nanoparticle size distribution, and (d) HRTEM image of Co/SiO_2.
Supplementary Figure 19. FT-IR spectra of SiO_2, Co/SiO_2, and Co@Si_x samples.
Supplementary Figure 20. (a) Co K-edge XANES spectra and (b) first-order derivative spectra of Co_3O_4@Si_x and Co_3O_4/SiO_2 samples.
Supplementary Figure 21. XAS spectra of O K-edges for various samples.
Supplementary Figure 22. XPS full spectrum of Co@$Si_{0.95}$.
Supplementary Figure 23. *In-situ* O *1s* XPS spectra of Co_3O_4@$Si_{0.95}$ under 0.1 mbar of H_2.
Supplementary Figure 24. (a–e) Co *2p* XPS spectra of various Co@Si_x catalysts. (f) The relationship between methanol yield with Co^0/Co^{2+} ratio in CO_2 hydrogenation over various

catalysts.

Supplementary Figure 25. H_2-TPR profiles of Co_3O_4, Co_3O_4@$Si_{0.52}$, Co_3O_4@$Si_{0.95}$, Co_3O_4@$Si_{1.48}$ and Co_3O_4@$Si_{1.87}$ samples.

Supplementary Figure 26. Co *2p* XPS spectra of (a) Co_3O_4 and (b–e) various Co_3O_4@Si_x catalysts.

Supplementary Figure 27. *In-situ* Co *2p* XPS spectra of Co_3O_4/SiO_2 in 0.1 mbar of H_2.

Supplementary Figure 28. *In-situ* O *1s* XPS spectra of Co_3O_4/SiO_2 in 0.1 mbar of H_2.

Supplementary Figure 29. H_2-TPR profile of Co_3O_4/SiO_2.

Supplementary Figure 30. (a) XRD pattern and (b) Co *2p* XPS spectrum of Co@$Si_{0.95}$ after high-pressure H_2 reduction (10% H_2 in Ar, 2.0 MPa) at 600 °C for 2 h.

Supplementary Figure 31. *In-situ* Raman spectra of (a) Co_3O_4, (b) Co_3O_4@$Si_{0.95}$, (c) Co_3O_4@$Si_{1.87}$ and (d) Co_3O_4/SiO_2 under H_2 reduction treatment.

Supplementary Figure 32. (a) XRD pattern and (b) Co *2p* XPS spectrum of Co@$Si_{0.95}$ after CO_2 hydrogenation for 100 h. The reactions are the same as those in Figure 2d in the main text.

Supplementary Figure 33. Data characterizing the performances of Co/SiO_2 and Co@$Si_{0.95}$ catalysts in H–D exchange test ($H_2 + D_2 = 2HD$).

Supplementary Figure 34. Scheme showing the different cobalt sites in Co@Si_x catalysts. For both the cobalt particles on the surface and embedded within the silica sheath, the bare cobalt species are Co^0 and the cobalt-silica interface has abundant $Co^{\delta+}$, which are highlighted by light blue and red arrows, respectively.

Supplementary Figure 35. Etching-XPS analysis of Co@$Si_{0.95}$ catalyst.

Supplementary Figure 36. Comparison of activity of Co@$Si_{0.95}$ and NaOH-treated Co@$Si_{0.95}$ (Co@$Si_{0.95}$-Na) catalysts in CO_2 hydrogenation.

Supplementary Figure 37. (a, b) TEM images, (c) Co nanoparticle size distribution, and (d) HRTEM image of Co@$Si_{0.95}$ after CO_2 hydrogenation for 100 h. The reaction conditions are the same as those in Figure 2d in the main text.

Supplementary Figure 38. XRD pattern of used Co/SiO_2 in CO_2 hydrogenation for 100 h. The reaction conditions are the same as those in Figure 2d in the main text.

Supplementary Figure 39. (a–c) HRTEM images and (d) Co nanoparticle size distribution of Co/SiO_2 after CO_2 hydrogenation for 100 h. The reaction conditions are the same as those in Figure 2d in the main text.

Supplementary Figure 40. *In-situ* DRIFTS spectra of various catalysts in flowing CO_2 at 250 °C.

Supplementary Figure 41. *In-situ* DRIFTS spectra of Co@Si_x catalysts at 350 °C with feed gases of CO_2 and H_2.

Supplementary Figure 42. *In-situ* DRIFTS spectra of CO_2 pretreated Co@$Si_{0.95}$ catalyst with pulsed H_2 at 350 °C.

Supplementary Figure 43. *In-situ* DRIFTS spectra of Co/SiO_2 in CO_2 hydrogenation at 350 °C (feed gases of CO_2 and $CO_2 + H_2$).

Supplementary Figure 44. *In-situ* (a) Co *2p* and (b) O *1s* XPS spectra of Co@$Si_{0.95}$ catalyst under 1.2 mbar CO_2 and H_2 atmosphere at 250 °C.

Supplementary Figure 45. *In-situ* Raman spectra of (a) CoO_x, (b–e) various Co@Si_x and (f) Co/SiO_2 catalysts under CO_2 and $CO_2 + H_2$ treatment at 250 °C.

Supplementary Figure 46. *In-situ* (a) Co *2p*, (b) O *1s*, and (c) C *1s* XPS spectra of Co/SiO_2 under 1.2 mbar of mixed CO_2 and H_2 with different ratios at 250 °C.

Supplementary Figure 47. *In-situ* (a) Co *2p*, (b) O *1s* and (c) C *1s* XPS spectra of Co@$Si_{0.95}$ under

mixed gases (1.0 mbar CO_2 and 0.1 mbar H_2) treatment for 5 min, and slowly extracted from the chamber at 250 °C.

Supplementary Figure 48. *In-situ* (a) Co *2p*, (b) O *1s* and (c) C *1s* XPS spectra of Co/SiO_2 mixed gases (1.0 mbar CO_2 and 0.1 mbar H_2) treatment for 5 min, and slowly extracted from the chamber at 250 °C.

Supplementary Figure 49. The kinetic plots of $Co@Si_{0.95}$ and Co/SiO_2 in CO_2 hydrogenation on the basis of (a) H_2 partial pressure and (b) CO_2 partial pressure.

Supplementary Figure 50. Arrhenius plots for CO, CH_3OH, and CH_4 formation over (a) $Co@Si_{0.95}$ and (b) Co/SiO_2 catalysts.

Supplementary Figure 51. Dependences of the CO conversion, products selectivity of the $Co@Si_{0.95}$ catalyst for CO hydrogenation.

Supplementary Figure 52. MeOH-TPSR profiles of (a) $Co@Si_{0.95}$ and (b) Co/SiO_2 catalysts.

Supplementary Table 1. Atomic ratio of Si/Co on several $Co@Si_x$ catalysts.

Supplementary Table 2. The surface area and volume data of various samples.

Supplementary Table 3. Comparison of the activities of various catalysts for the CO_2 hydrogenation to methanol.

Supplementary Table 4. EXAFS structure parameters representing $Co@Si_x$ and Co/SiO_2 samples.

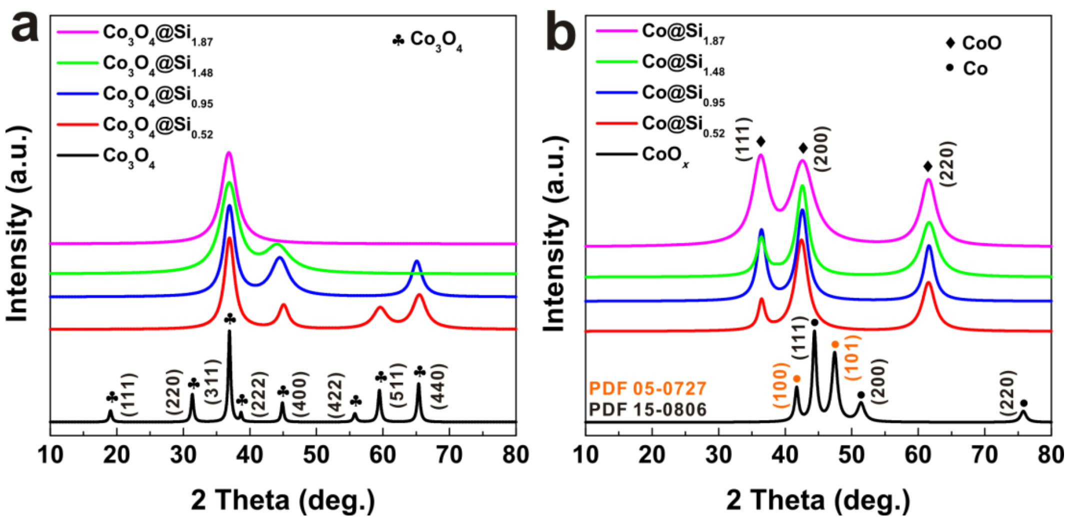

Supplementary Figure 1. XRD patterns of (a) Co_3O_4@Si_x and (b) Co@Si_x catalysts.

Note: All Co_3O_4@Si_x samples have Co_3O_4 as a dominant phase. After reduction at 600 °C, the CoO_x without silica was transformed into metallic Co (41.6°, 44.2°, 47.5°, 51.5° and 75.9°). Under the equivalent reduction treatment, the Co@Si_x samples still exhibited CoO as the dominant phase as firmed by the peaks at 36.4°, 42.5° and 61.5°.

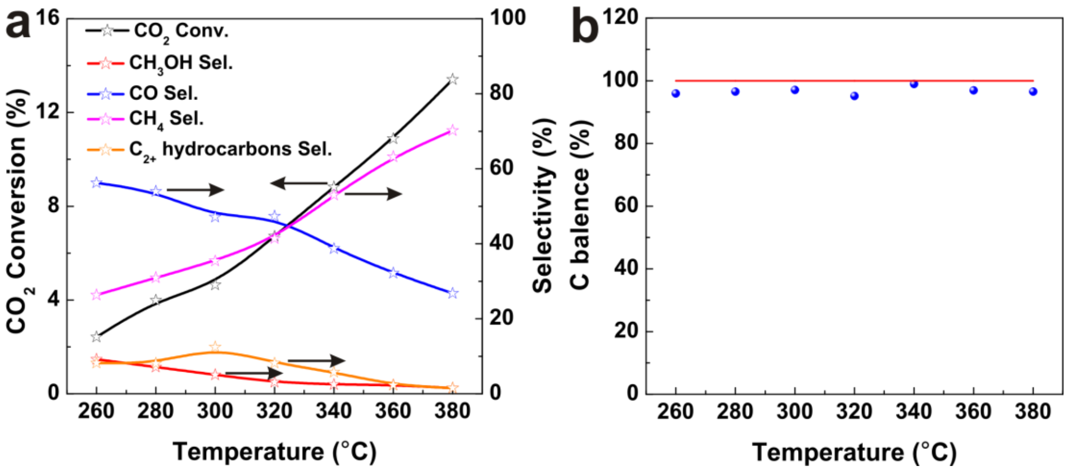

Supplementary Figure 2. (a) Dependences of the CO_2 conversion, product selectivity, and (b) carbon balance on temperature over CoO_x catalyst. Reaction conditions: 0.1 g of catalyst, 2.0 MPa, $H_2/CO_2 = 3:1$, GHSV = 12000 mL/g h.

Note: The lower C_{2+} selectivity than the general Fischer–Tropsch process should be due to the abundant CO_2 and scarce CO in the mixed gas.

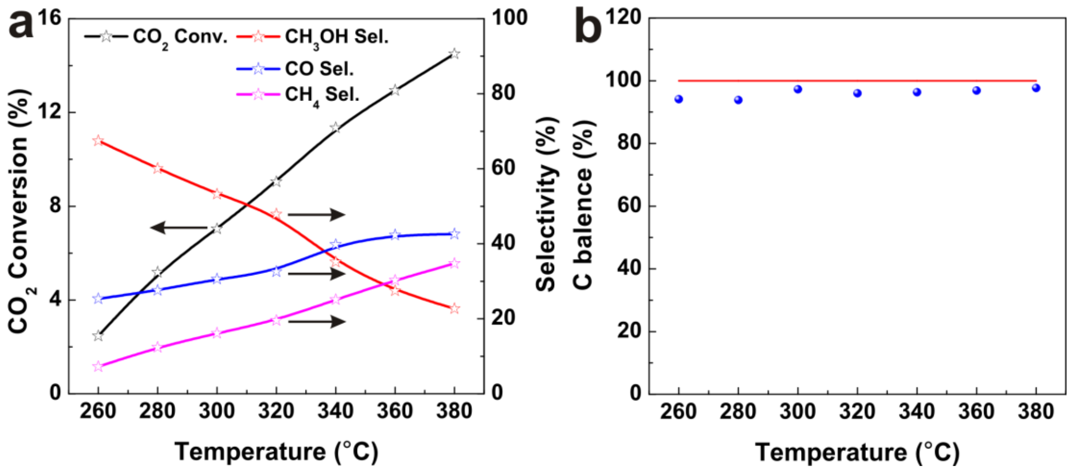

Supplementary Figure 3. (a) Dependences of the CO_2 conversion, product selectivity, and (b) carbon balance on temperature over Co@$Si_{0.52}$ catalyst. Reaction conditions: 0.15 g of catalyst, 2.0 MPa, $H_2/CO_2 = 3{:}1$, GHSV = 8000 mL/g h.

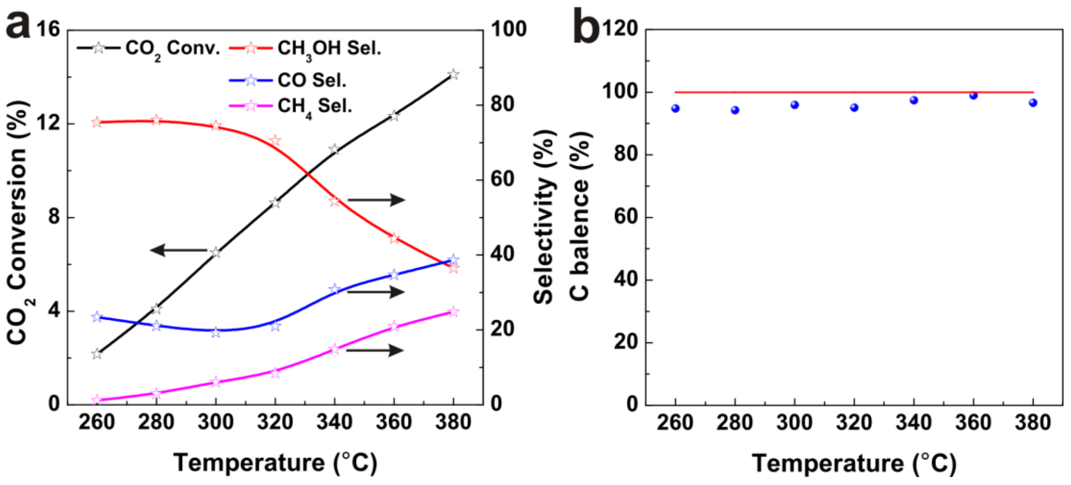

Supplementary Figure 4. (a) Dependences of the CO_2 conversion, product selectivity, and (b) carbon balance on temperature over Co@$Si_{0.95}$ catalyst. Reaction conditions: 0.2 g of catalyst, 2.0 MPa, $H_2/CO_2 = 3:1$, GHSV = 6000 mL/g h.

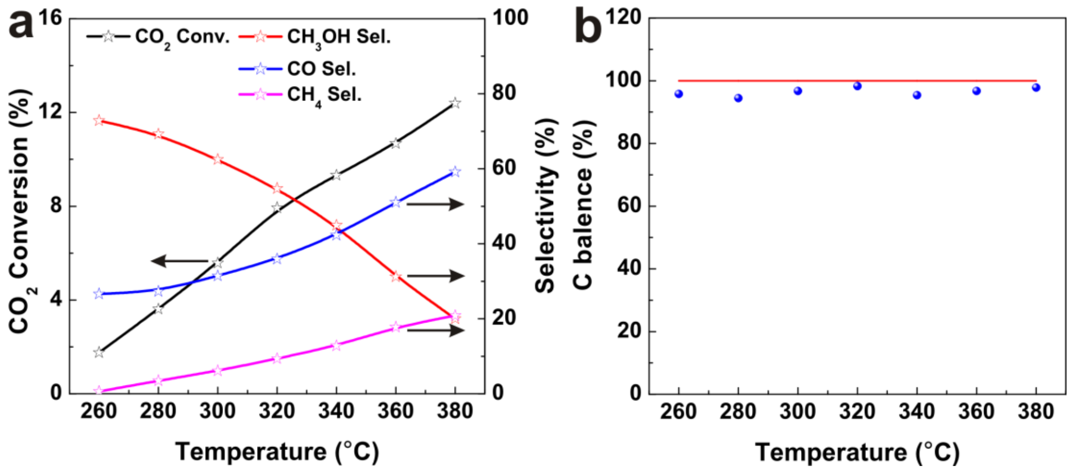

Supplementary Figure 5. (a) Dependences of the CO_2 conversion, product selectivity, and (b) carbon balance on temperature over Co@$Si_{1.48}$ catalyst. Reaction conditions: 0.25 g of catalyst, 2.0 MPa, H_2/CO_2 = 3:1, GHSV = 4800 mL/g h.

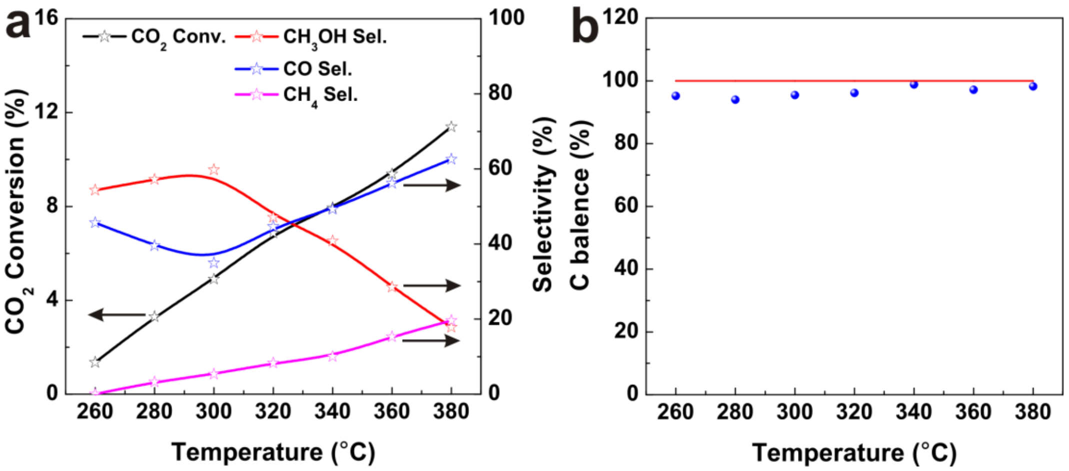

Supplementary Figure 6. (a) Dependences of the CO_2 conversion, product selectivity, and (b) carbon balance on temperature over Co@Si$_{1.87}$ catalyst. Reaction conditions: 0.3 g of catalyst, 2.0 MPa, H_2/CO_2 = 3:1, GHSV = 4000 mL/g h.

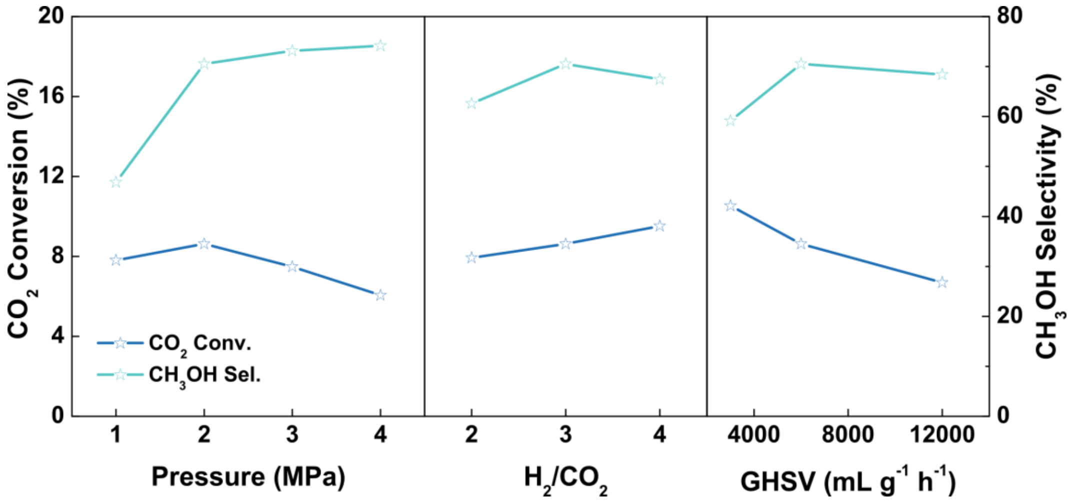

Supplementary Figure 7. Data characterizing the influences of reaction pressure, H_2/CO_2 ratio, and velocity to the performances of Co@$Si_{0.95}$ catalyst in CO_2 hydrogenation. Standard reaction conditions: 0.2 g of Co@$Si_{0.95}$ catalyst, 2.0 MPa, H_2/CO_2 = 3:1, 320 °C, GHSV = 6000 mL/g h.

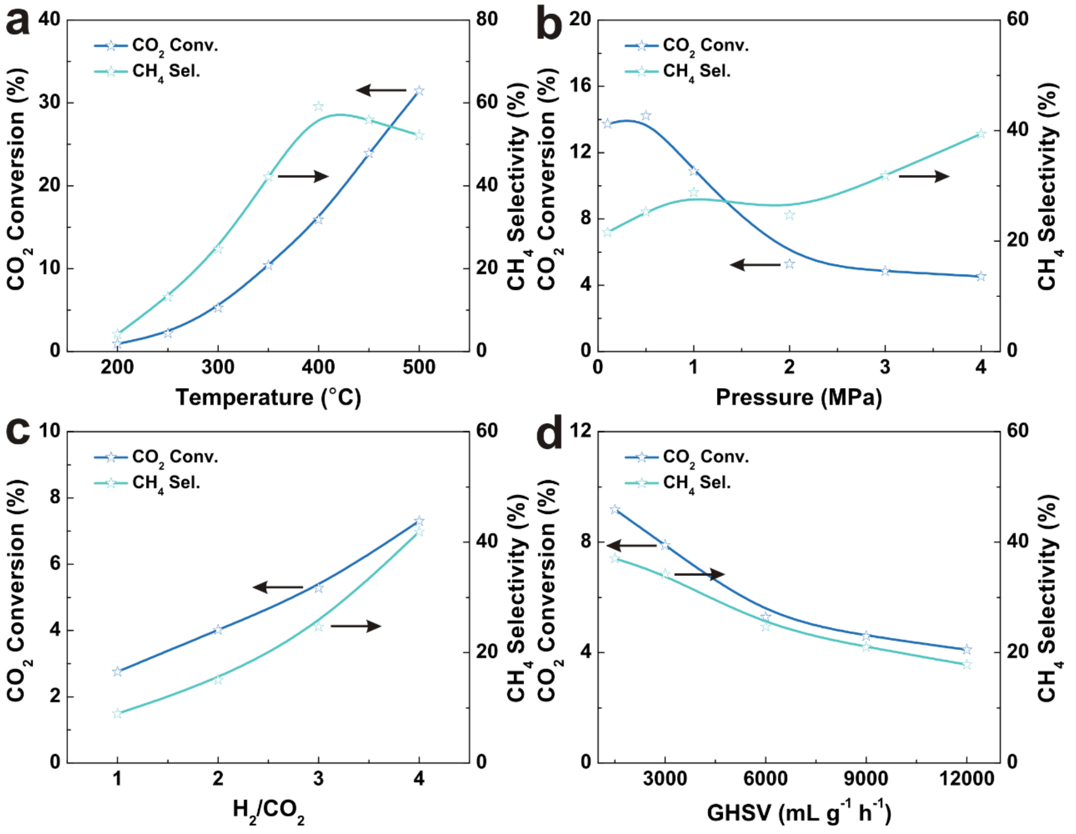

Supplementary Figure 8. Data characterizing the CO_2 conversions and CH_4 selectivity on (a) reaction temperature, (b) pressure, (c) H_2/CO_2 ratio, and (d) velocity over Co/SiO_2 catalyst. Reaction conditions: 0.2 g of catalyst, 2.0 MPa, H_2/CO_2 = 3:1, 300 °C, GHSV = 6000 mL/g h.

Note: Methane is usually formed on the cobalt catalysts in CO_2 hydrogenation, but the methane and CO selectivities depend on the multiple factors (e.g. CO_2 conversion, reaction temperature, pressure, catalyst amount, and the support)[1–10]. In our experiments, we found that the Co/SiO_2 and CoO_x samples with metallic cobalt species exhibited higher methane selectivity than the $Co@Si_x$ catalyst in CO_2 hydrogenation. In these cases, the methane selectivity is still slightly lower than CO (Figure 2a), which should be due to the employed reaction conditions, and similar phenomena have been observed previously[1,4,7,10].

We explored the methane selectivity as a function of reaction temperature in the Co/SiO_2 and CoO_x catalyzed CO_2 hydrogenation. As shown in Supplementary Figures 2 and 10, the methane selectivity remarkably increased with the reaction temperatures. For example, the Co/SiO_2 gave CH_4 selectivity at 24.7% at 300 °C. By increasing the reaction temperature to 380 °C, the CH_4 selectivity raised to 54.9%. For the CoO_x catalyst, the CH_4 selectivity was 35.4% at 300 °C and then increased to 70.2% at 380 °C. Further increasing the reaction temperature to 500 °C gave lower CH_4 selectivity at 52.2% on the Co/SiO_2 catalyst (Supplementary Figure 8a). This might be attributed to the strong exothermic nature of the CO_2 methanation, leading to thermodynamically favorable CO formation *via* reverse water–gas shift reaction and decrease of CH_4

selectivity[11]. These data suggest that the reaction temperatures have a significant effect on the CH_4 and CO selectivity[4,5,10]. In addition to the reaction temperature, we also explored the influences of reaction pressure, feed gas composition, and catalyst amount to the methane selectivity in CO_2 hydrogenation (Supplementary Figures 8b–d). The higher reaction pressure, more hydrogen feed, and larger catalyst amount (low GHSV) are regarded to be the factors for enhancing the methane selectivity.

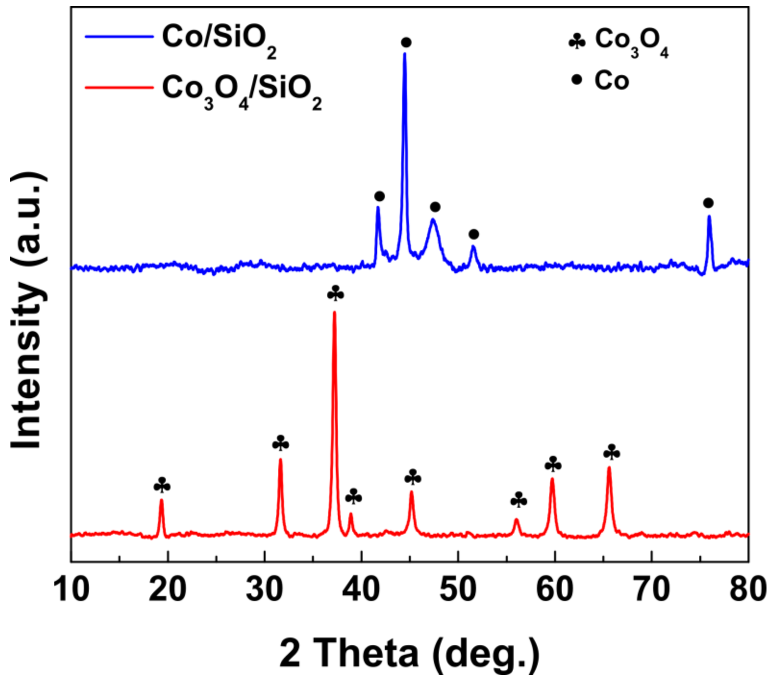

Supplementary Figure 9. XRD patterns of as-synthesized Co_3O_4/SiO_2 and Co/SiO_2.

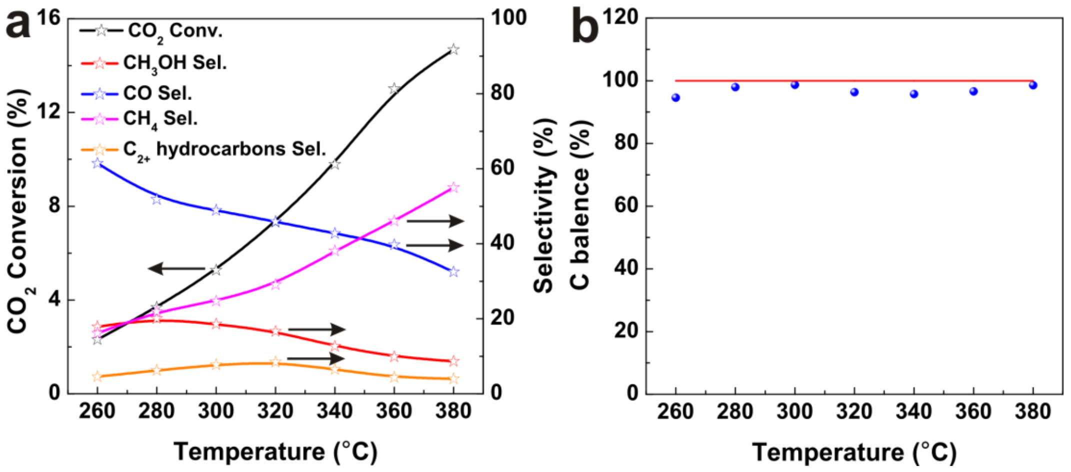

Supplementary Figure 10. (a) Dependences of the CO_2 conversion, products selectivity, and (b) carbon balance on temperature over Co/SiO_2 catalyst. Reaction conditions: 0.2 g of catalyst, 2.0 MPa, H_2/CO_2 = 3:1, GHSV = 6000 mL/g h.

Note: The lower C_{2+} selectivity than the general Fischer–Tropsch process should be due to the abundant CO_2 and scarce CO in the mixed gas.

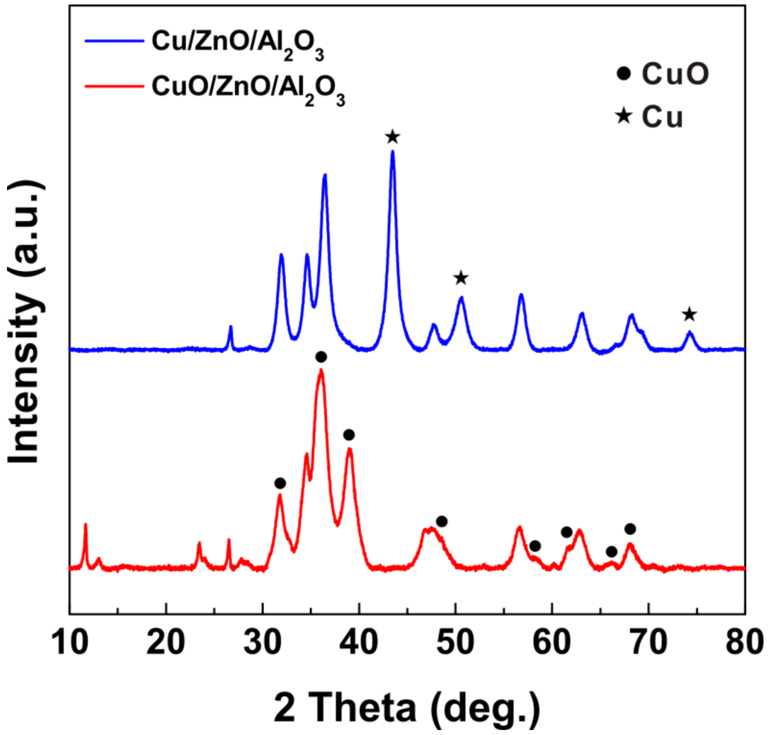

Supplementary Figure 11. XRD patterns of $CuO/ZnO/Al_2O_3$ and $Cu/ZnO/Al_2O_3$ catalysts.

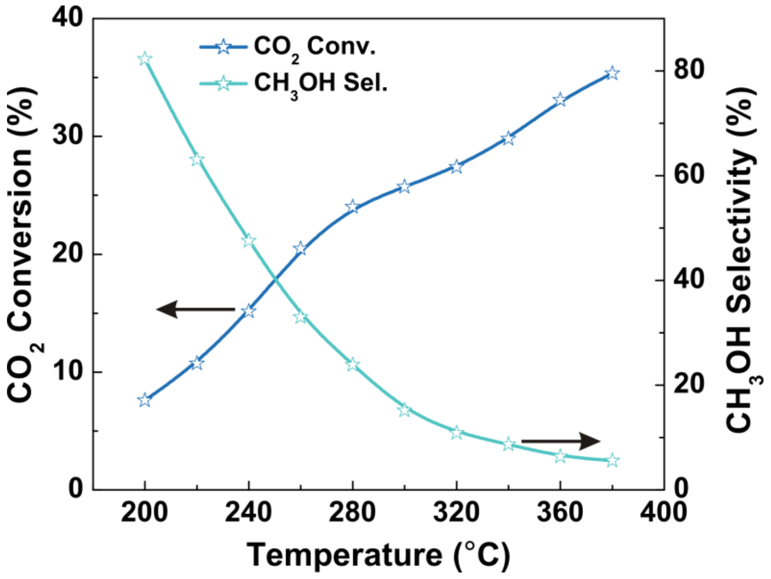

Supplementary Figure 12. (a) Dependences of the CO_2 conversion, products selectivity, and (b) carbon balance on temperature over Cu/ZnO/Al_2O_3 catalyst. Reaction conditions: 0.2 g of catalyst, 2.0 MPa, H_2/CO_2 = 3:1, GHSV = 6000 mL/g h.

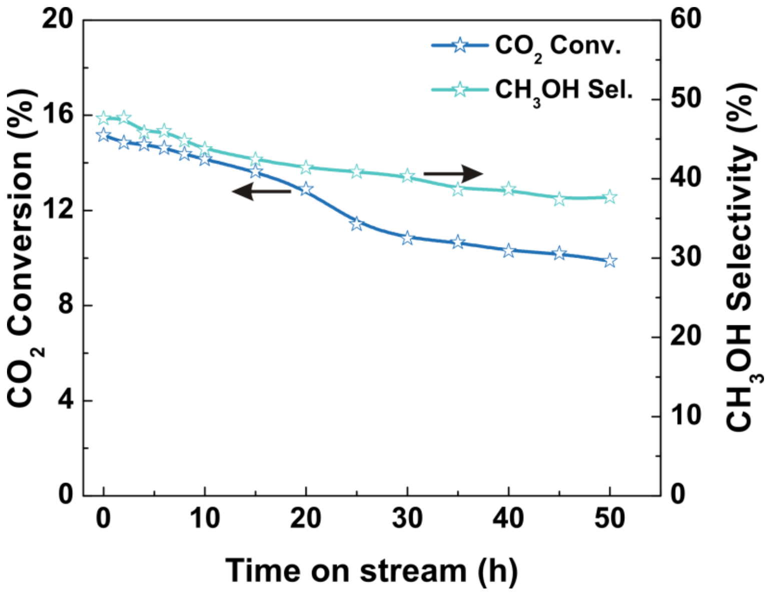

Supplementary Figure 13. Data showing the durability of Cu/ZnO/Al_2O_3 catalyst in CO_2 hydrogenation. Reaction conditions: 0.2 g of catalyst, 2.0 MPa, H_2/CO_2 = 3:1, 240 °C, GHSV = 6000 mL/g h.

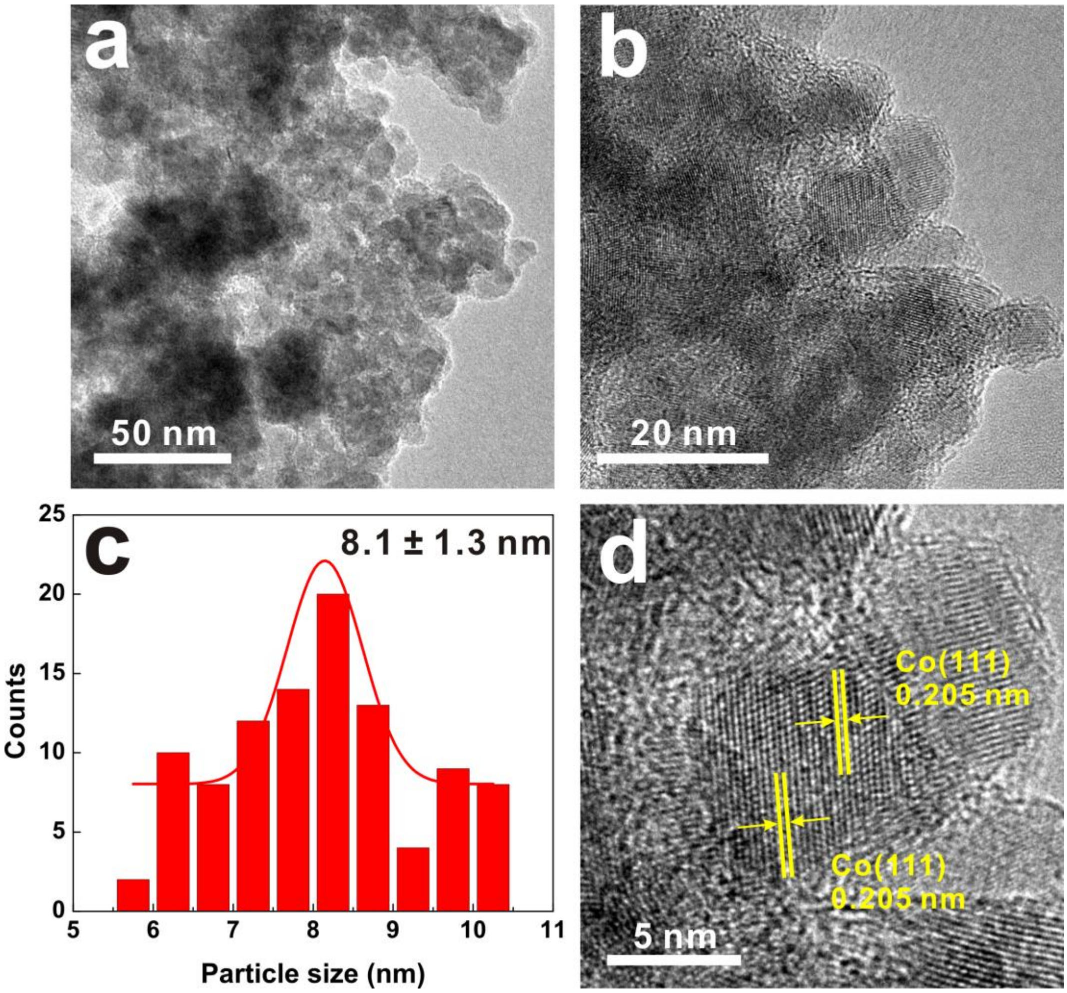

Supplementary Figure 14. (a, b) TEM images, (c) Co nanoparticle size distribution, and (d) HRTEM image of CoO_x.

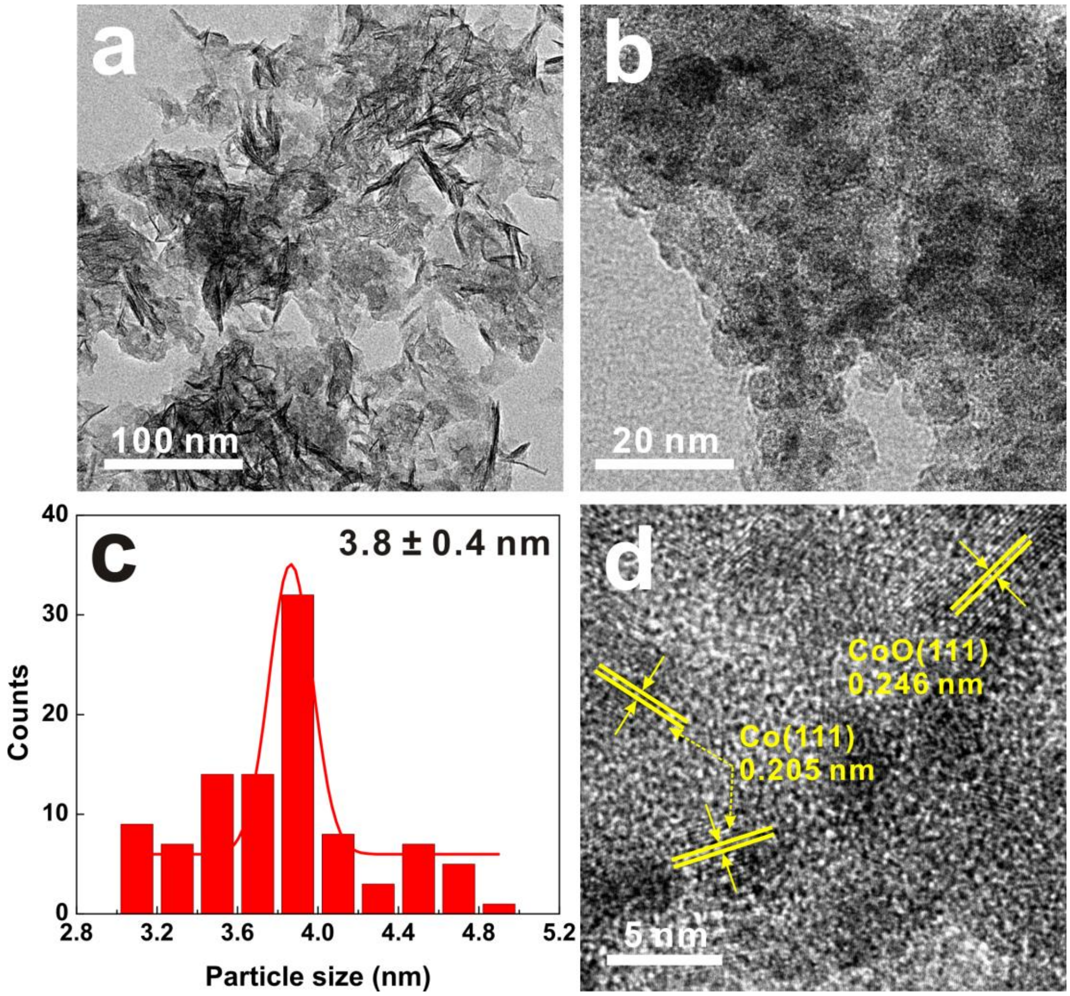

Supplementary Figure 15. (a, b) TEM images, (c) Co nanoparticle size distribution, and (d) HRTEM image of $Co@Si_{0.52}$.

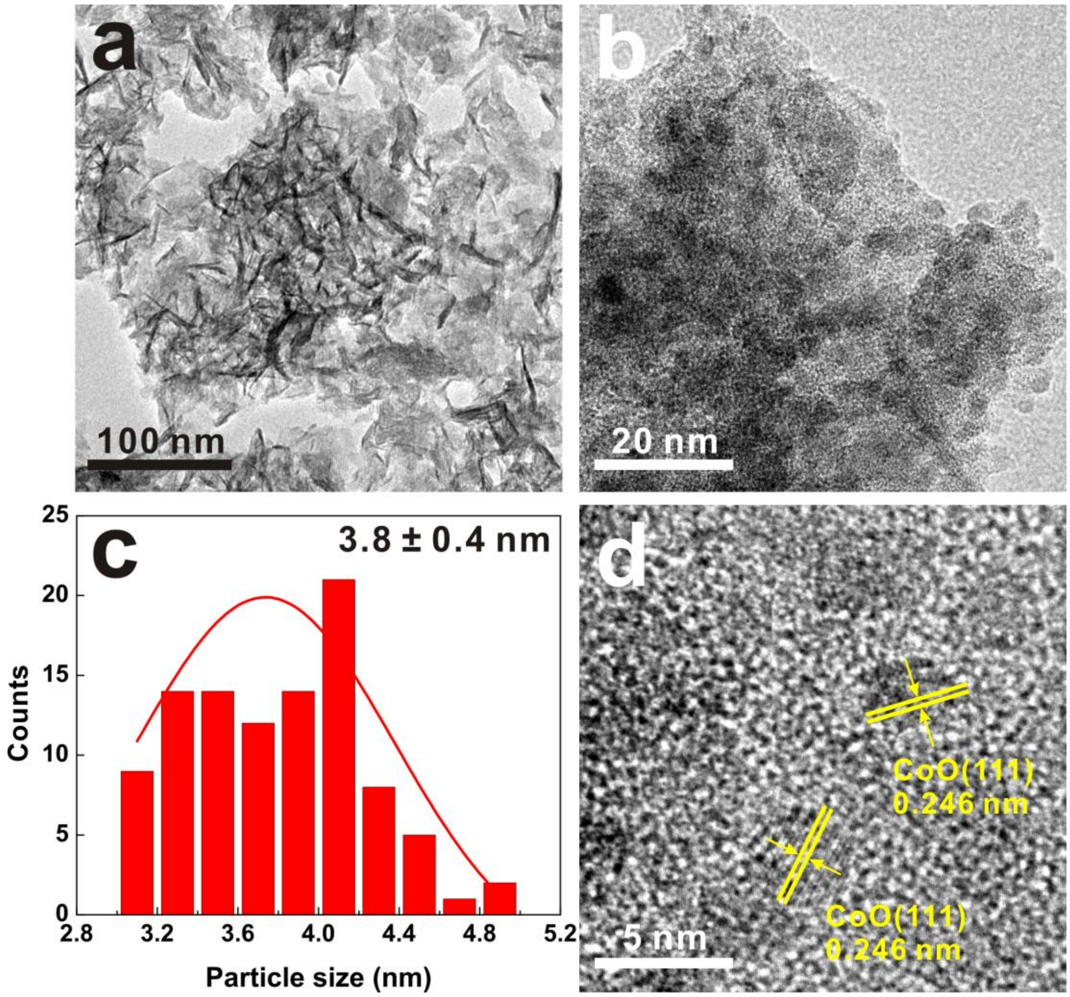

Supplementary Figure 16. (a, b) TEM images, (c) Co nanoparticle size distribution, and (d) HRTEM image of Co@$Si_{1.48}$.

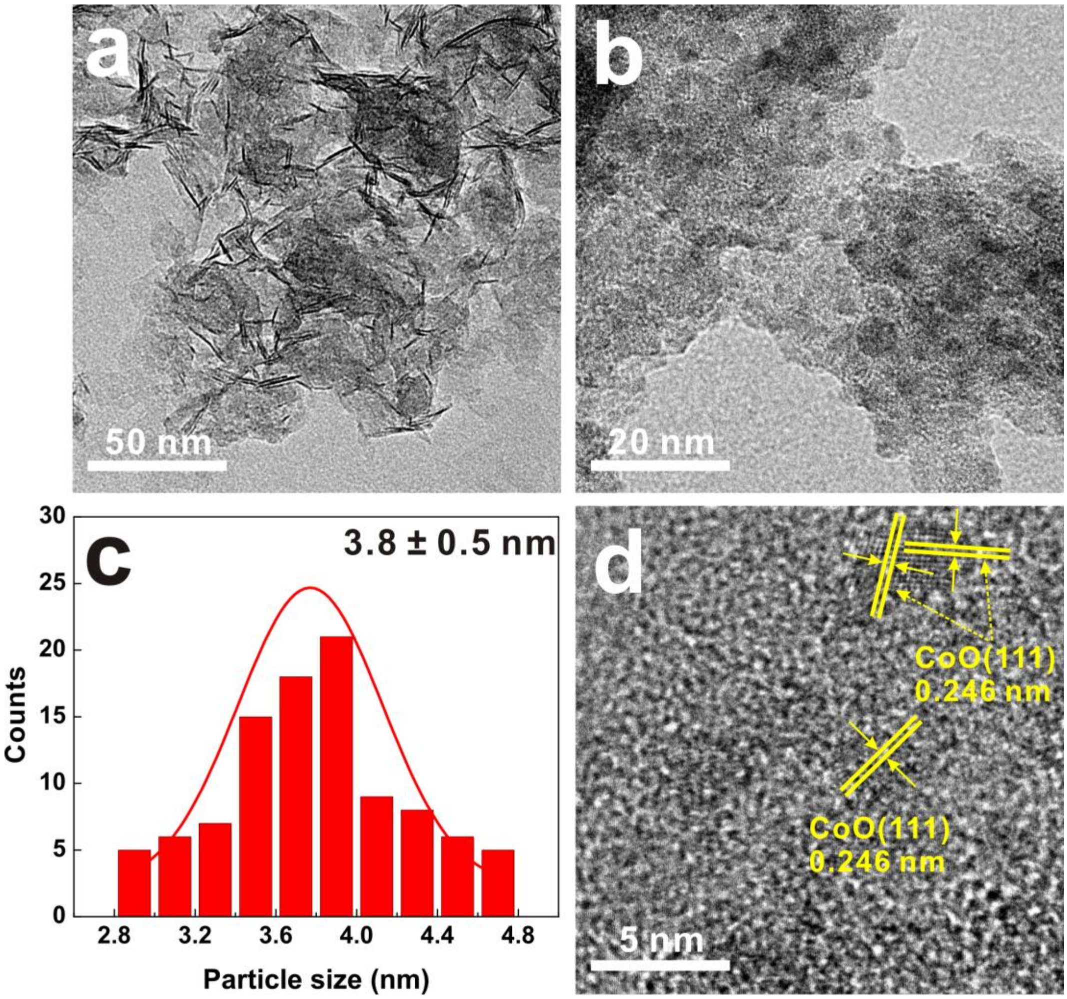

Supplementary Figure 17. (a, b) TEM images, (c) Co nanoparticle size distribution, and (d) HRTEM image of Co@$Si_{1.87}$.

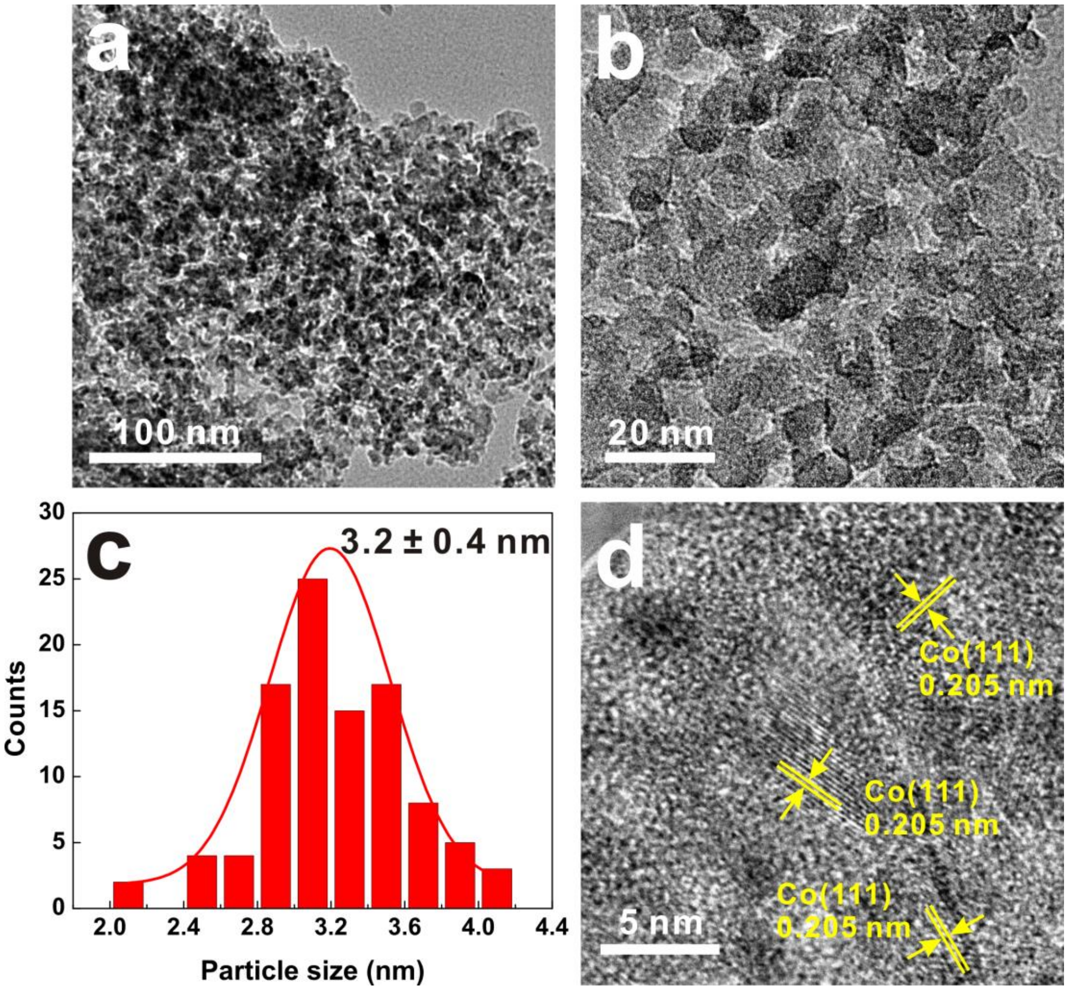

Supplementary Figure 18. (a, b) TEM images, (c) Co nanoparticle size distribution, and (d) HRTEM image of Co/SiO_2.

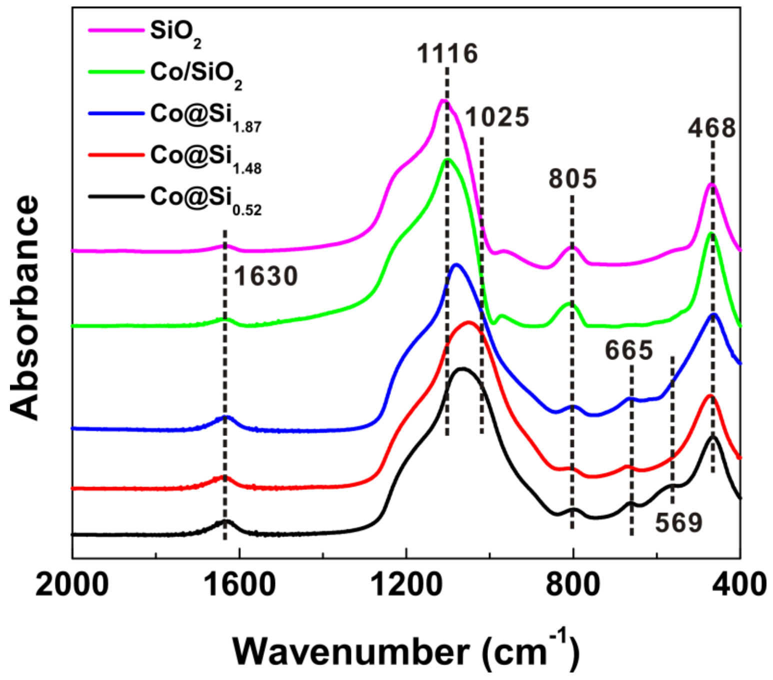

Supplementary Figure 19. FT-IR spectra of SiO_2, Co/SiO_2, and Co@Si$_x$ samples.

Note: FT-IR spectroscopy was employed to identify the cobalt oxides and cobalt phyllosilicates species. The band at 665 cm^{-1} can be assigned to the Co–O stretching of Co–O–Si bonds in cobalt phyllosilicates, which is also confirmed by the presence of another band at 569 cm^{-1}. Besides, the structural hydroxyl groups of cobalt phyllosilicates are observed at 1025 cm^{-1}. Strong absorption at 1116, 805 and 468 cm^{-1} indicates the silica network, while slight band at 1630 cm^{-1} is assigned to the O–H bending vibration of the adsorbed water[12,13].

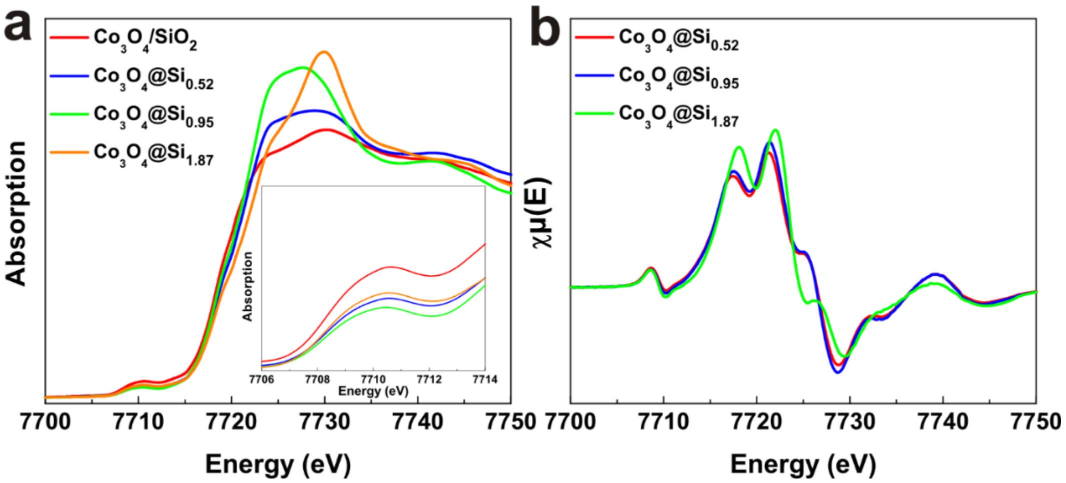

Supplementary Figure 20. (a) Co K-edge XANES and (b) first-order derivative spectra of $Co_3O_4@Si_x$ and Co_3O_4/SiO_2 samples.

Note: All of $Co_3O_4@Si_x$ and Co_3O_4/SiO_2 samples exhibit a pre-edge feature at 7710.4 eV and the first-order derivative peaks at 7717.5 eV and 7721.5 eV, which are similar to the pre-edge features of Co_3O_4 and the edge positions of Co^{2+} and Co^{3+} cations, respectively[14,15].

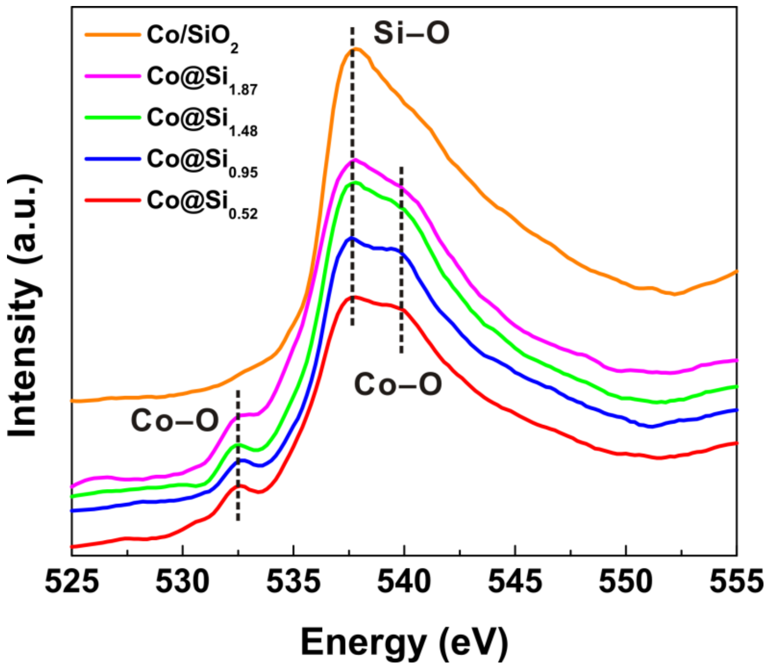

Supplementary Figure 21. XAS spectra of O K-edges for various samples.

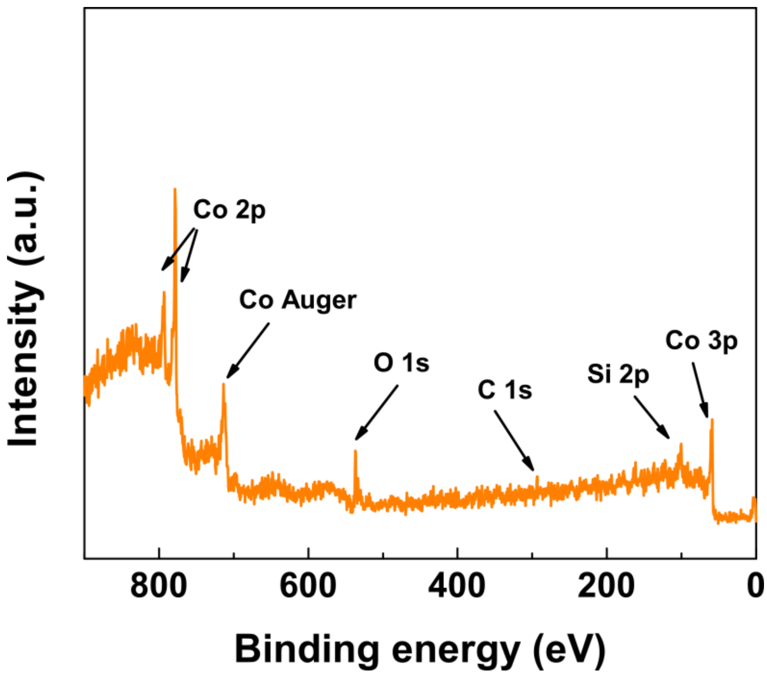

Supplementary Figure 22. XPS full spectrum of Co@$Si_{0.95}$.

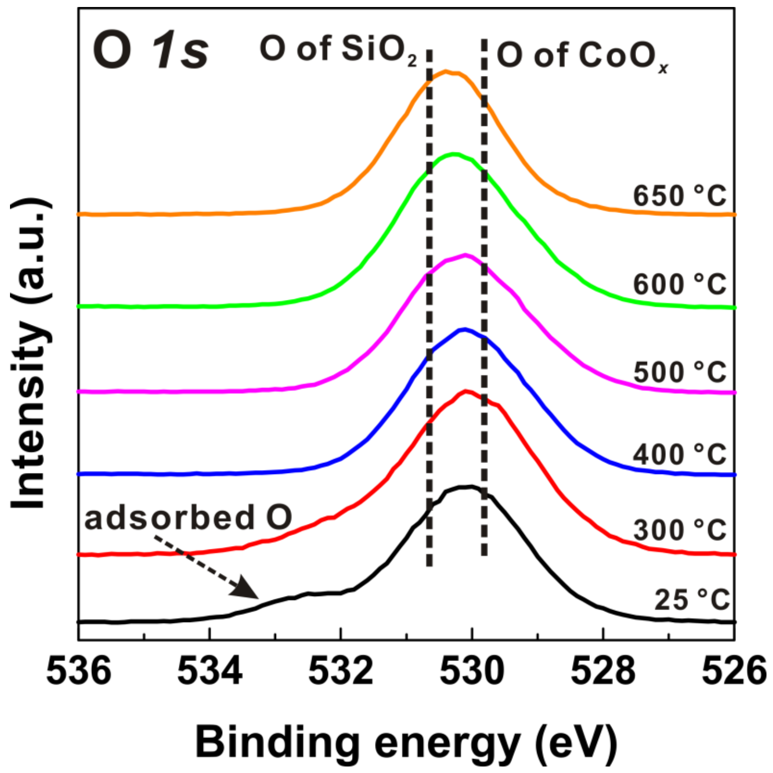

Supplementary Figure 23. *In-situ* O *1s* XPS spectra of Co_3O_4@$Si_{0.95}$ under 0.1 mbar of H_2.

Note: The oxygen of SiO_2 and CoO_x are located at 529.8 and 530.6 eV in O *1s* XPS spectra, respectively. Increasing the reduction temperature led to the shift of O *1s* peak to higher binding energies, because part of the oxygen of CoO_x is eliminated and the oxygen of SiO_2 (including the O of Co–O–Si) is stable.

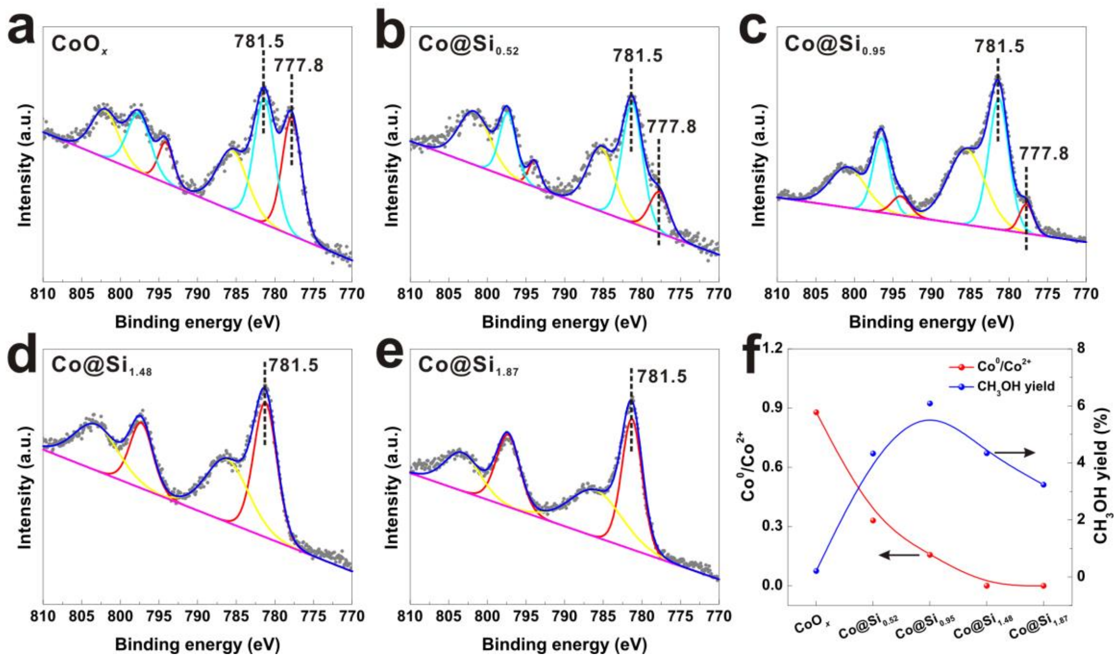

Supplementary Figure 24. (a–e) Co *2p* XPS spectra of various Co@Si$_x$ catalysts. (f) The relationship between methanol yield with Co^0/Co^{2+} ratio in CO_2 hydrogenation over various catalysts.

Note: The Co *2p* XPS spectra of CoO$_x$, Co@Si$_{0.52}$ and Co@Si$_{0.95}$ show obvious peaks at 781.5 and 777.8 eV, assigned to Co^{2+} and Co^0, respectively. Only the peak at 781.5 eV is observed over Co@Si$_{1.48}$ and Co@Si$_{1.87}$ samples.

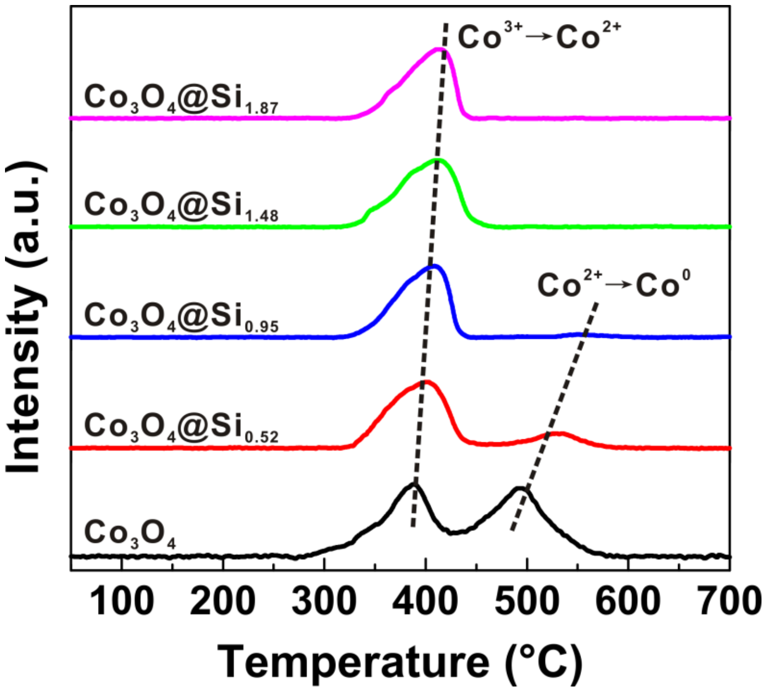

Supplementary Figure 25. H_2-TPR profiles of Co_3O_4, Co_3O_4@$Si_{0.52}$, Co_3O_4@$Si_{0.95}$, Co_3O_4@$Si_{1.48}$ and Co_3O_4@$Si_{1.87}$ samples.

Note: The Co_3O_4 without silica species showed the reduction peaks at 387 and 494 ºC, which are assigned to the reduction of Co^{3+} to Co^{2+} and Co^{2+} to Co^0. When the cobalt oxide was modified by silica, these peaks were remarkably shifted to higher temperature and the Co^{2+} to Co^0 signal was weakened. For example, the Co_3O_4@$Si_{0.52}$ showed the Co^{3+} to Co^{2+} signal at 402 ºC and weak Co^{2+} to Co^0 signal at 533 ºC. The sample with lower Co/Si ratio, such as the Co_3O_4@$Si_{0.95}$ sample, exhibited even higher Co^{3+} to Co^{2+} signal at 410 ºC and almost undetectable Co^{2+} to Co^0 signal. These data demonstrate the different silica amount strongly influences the oxidation state of cobalt nanoparticles.

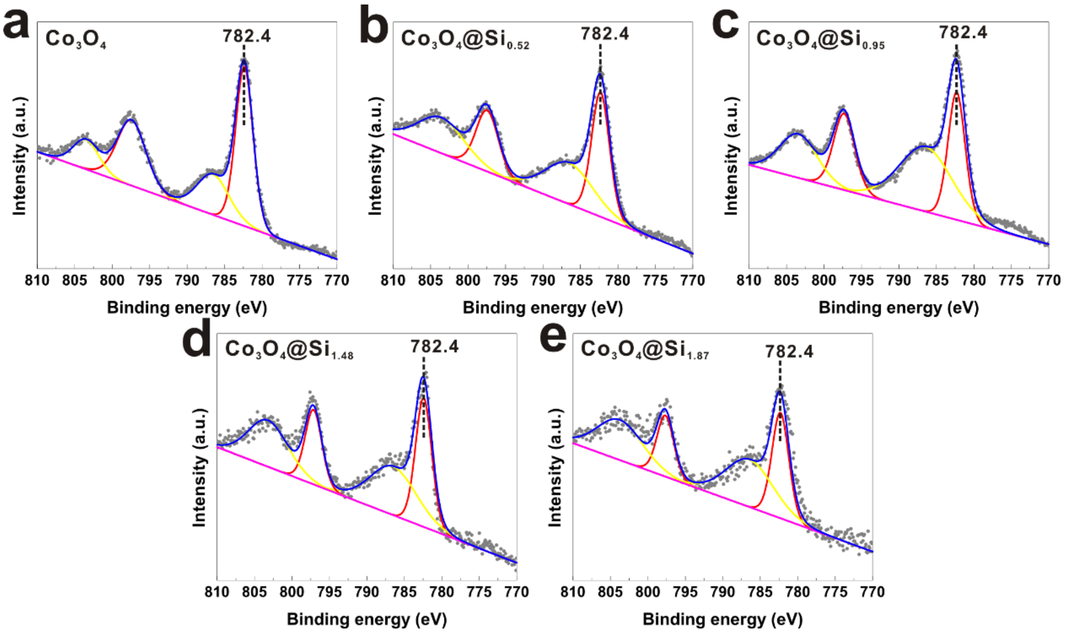

Supplementary Figure 26. Co *2p* XPS spectra of (a) Co_3O_4 and (b–e) various $Co_3O_4@Si_x$ catalysts.

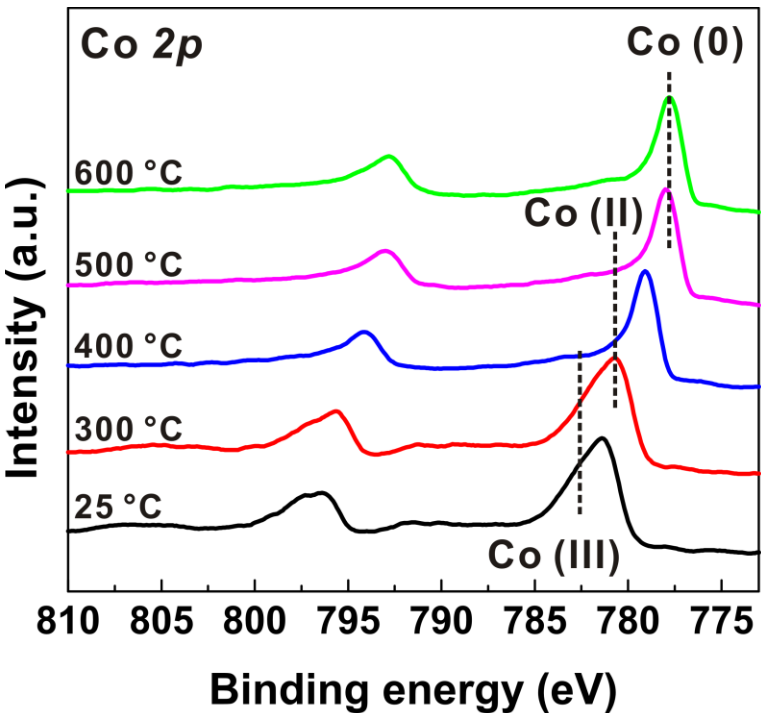

Supplementary Figure 27. *In-situ* Co *2p* XPS spectra of Co_3O_4/SiO_2 in 0.1 mbar of H_2.

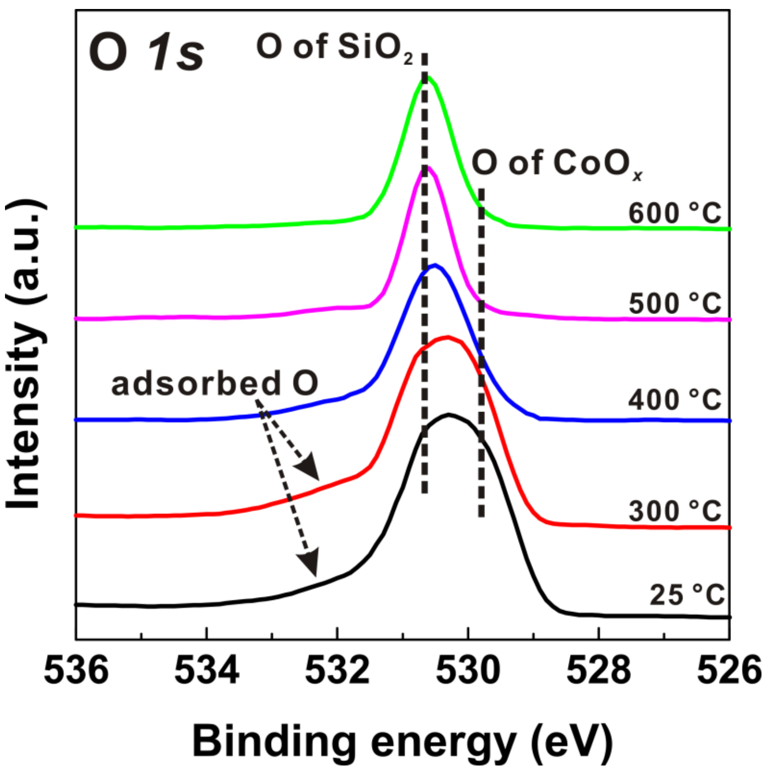

Supplementary Figure 28. *In-situ* O *1s* XPS spectra of Co_3O_4/SiO_2 in 0.1 mbar of H_2.

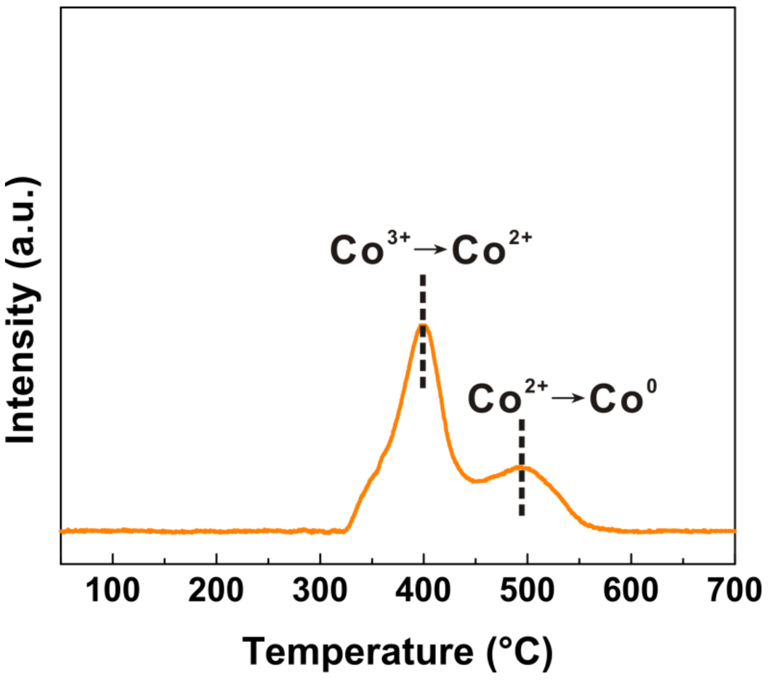

Supplementary Figure 29. H_2-TPR profile of Co_3O_4/SiO_2.

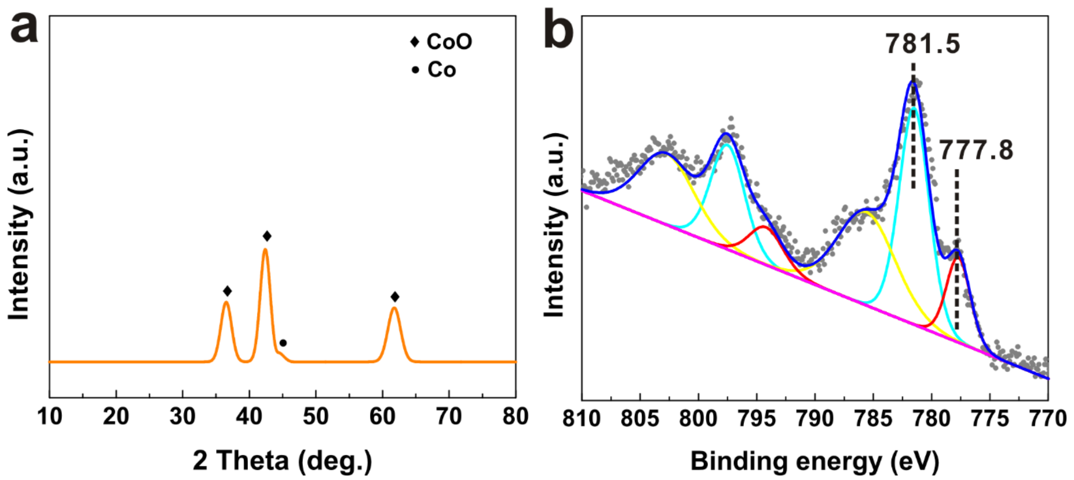

Supplementary Figure 30. (a) XRD pattern and (b) Co *2p* XPS spectrum of Co@$Si_{0.95}$ after high-pressure H_2 reduction (10% H_2 in Ar, 2.0 MPa) at 600 °C for 2 h.

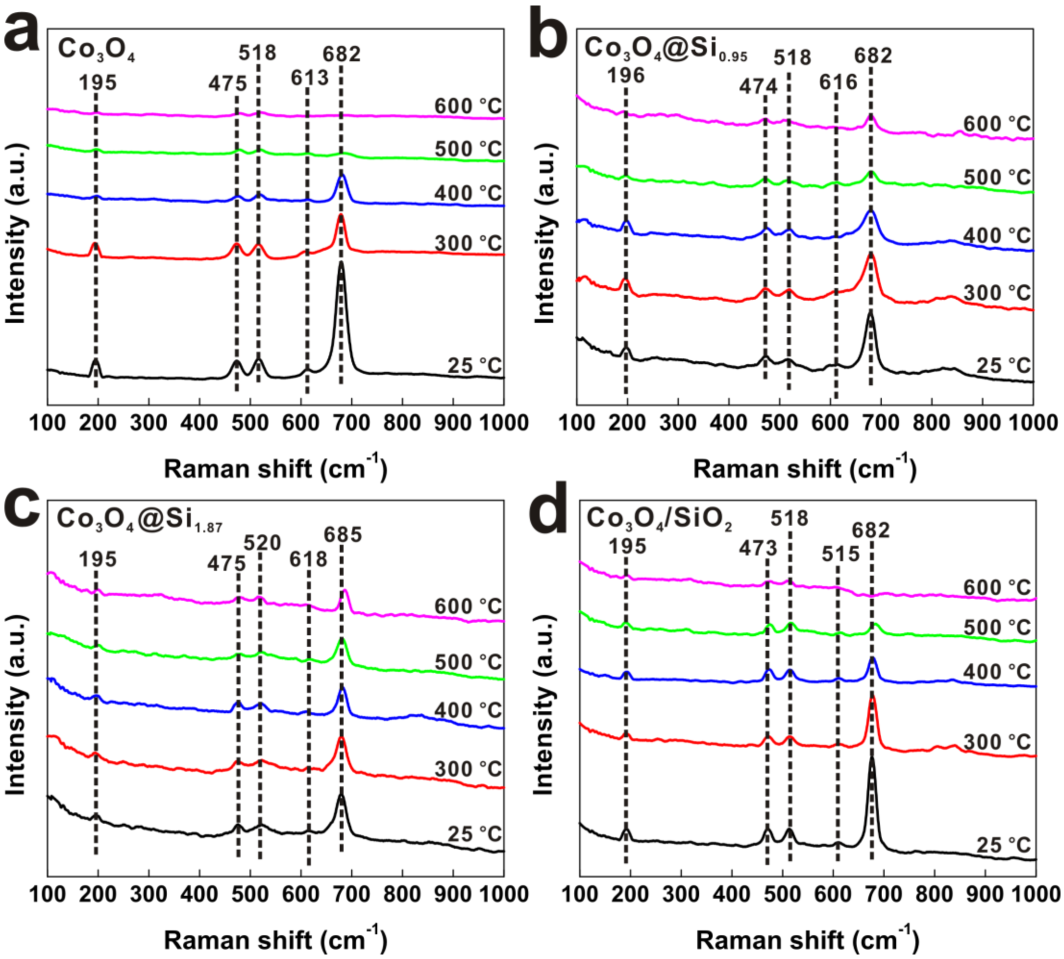

Supplementary Figure 31. *In-situ* Raman spectra of (a) Co_3O_4, (b) $Co_3O_4@Si_{0.95}$, (c) $Co_3O_4@Si_{1.87}$ and (d) Co_3O_4/SiO_2 under H_2 reduction treatment.

Note: *In-situ* Raman spectra of catalysts were performed under H_2 reduction treatment, which provided clear identification of the interaction between Co and O species. Co_3O_4, $Co_3O_4@Si_{0.95}$, $Co_3O_4@Si_{1.87}$, and Co_3O_4/SiO_2 all gave typical Raman peaks at 196, 474, 518, 616 and 682 cm^{-1} assigning to the Co–O species[16,17]. By reduction at higher temperatures, these peaks were reduced in intensity and even undetectable on Co_3O_4 and Co_3O_4/SiO_2 after reaction at 600 °C. But the $Co@Si_{0.95}$ and $Co@Si_{1.87}$ samples still exhibited these peaks under the equivalent treatments. These phenomena indicate the silica modification improves the anti-reduction ability of Co–O species, which is in good agreement with the results of XPS spectra.

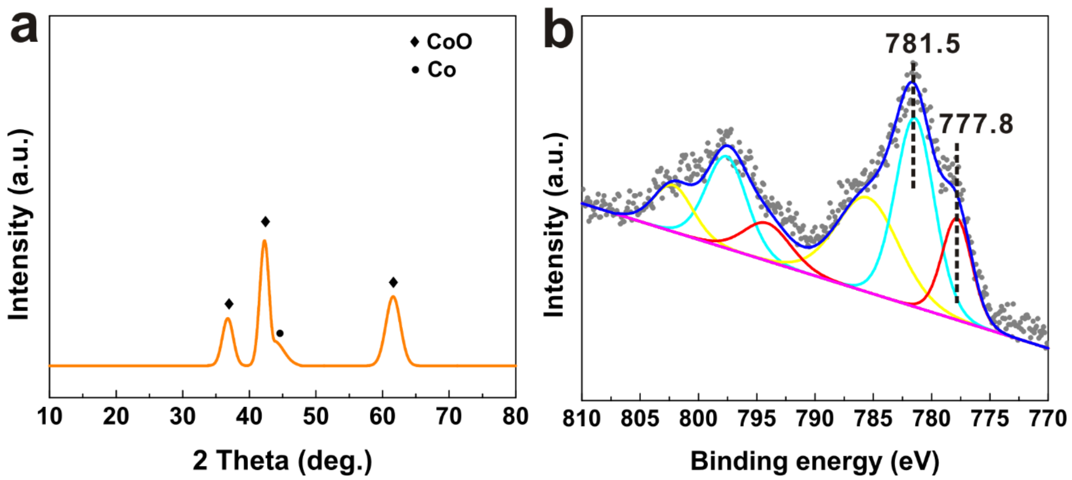

Supplementary Figure 32. (a) XRD pattern and (b) Co *2p* XPS spectrum of Co@$Si_{0.95}$ after CO_2 hydrogenation for 100 h. The reactions are the same as those in Figure 2d in the main text.

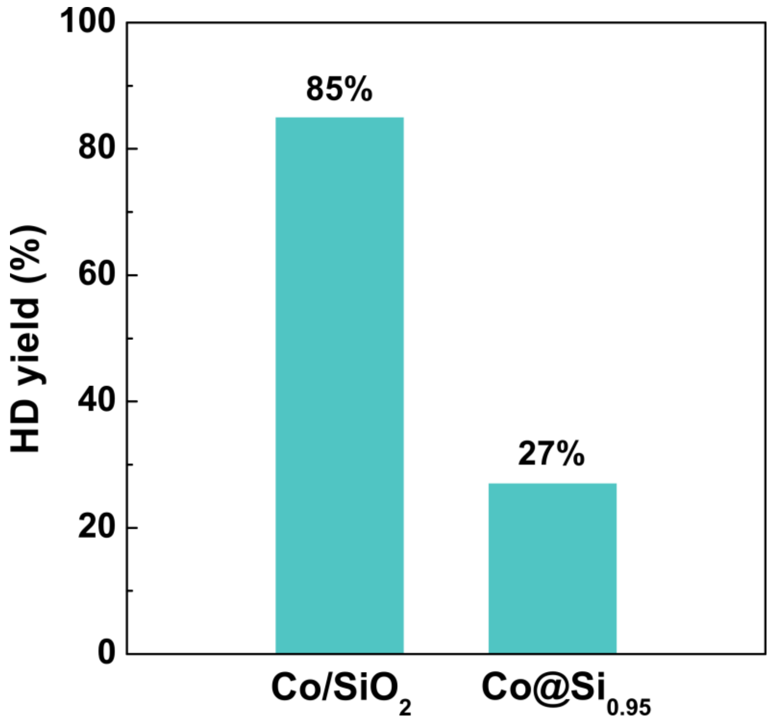

Supplementary Figure 33. Data characterizing the performances of Co/SiO_2 and Co@$Si_{0.95}$ catalysts in H–D exchange test (H_2 + D_2 = 2HD).

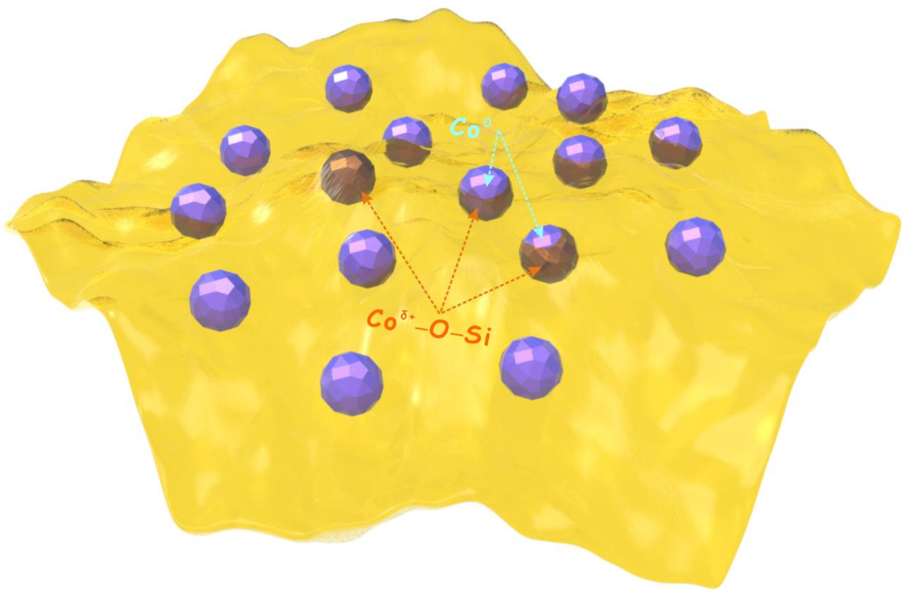

Supplementary Figure 34. Scheme showing the different cobalt sites in Co@Si$_x$ catalysts. For both the cobalt particles on the surface and embedded within the silica sheath, the bare cobalt species are Co^0 and the cobalt-silica interface has abundant $Co^{\delta+}$, which are highlighted by light blue and red arrows, respectively.

Note: After reduction with hydrogen, part of the cobalt species could be reduced into metallic cobalt, and the cobalt species interacting with silica still maintained the oxidation state because of the Co–O–SiO$_x$ interaction. According to our characterization data and the previous investigations on the copper phyllosilicates-derived catalysts[18–20]. The Co@Si$_x$ catalysts should have two types of cobalt species, including the metallic cobalt on the cobalt surface and CoO on the cobalt–silica interface. No matter the cobalt nanoparticles on the surface or within the amorphous sheath, the Co interacting with silica should be Co–O–SiO$_x$ and the bare surface should be Co^0. The scheme showing the catalyst structure is given in Supplementary Figure 34.

The M–O–SiO$_x$ interface have been widely investigated in hydrogenation reactions. Although the role of such interface has not been fully understood, the synergy of the metallic phase and oxide phase are regarded to be crucial. Generally, the metal catalyzed hydrogenation processes involve the steps including (i) dissociation of hydrogen, (ii) adsorption of unsaturated compounds, (iii) stepwise hydrogenation with H atoms. According to such knowledge, the metallic phase (e.g. Co^0) might activate H_2 and the oxide phase (e.g. $Co^{\delta+}$ species) adsorbs CO_2. This proposed pathway is in good agreement with the general knowledge on the Cu–O–SiO$_x$ catalysts[18,19]. In addition, the previous study has revealed that the Cu–O(H)–SiO$_x$ interface accelerate the hydrogen activation/splitting[20]. In order to identify whether the Co@Si$_x$ catalysts have similar feature, we removed part of the silanol groups by treating the sample with NaOH. The resulted Co@Si$_{0.95}$-Na catalyst exhibited remarkably reduced hydrogenation activity than the untreated Co@Si$_{0.95}$ (Supplementary Figure 36), suggesting the important role of Co–O–SiO$_x$ interface for the reaction. Possibly, both the metallic Co^0 and interfacial

$Co^{\delta+}$–O–SiO_x activated the hydrogen and accelerate the hydrogenation reactions. The interfacial $Co^{\delta+}$–O–SiO_x species stabilized the crucial reaction intermediates (e.g. *CH_3O) and improved the methanol selectivity, while the metallic Co^0 would catalyze the *CH_3O decomposition to form CO and methane. By optimizing the composition, the best catalyst was realized as Co@$Si_{0.95}$.

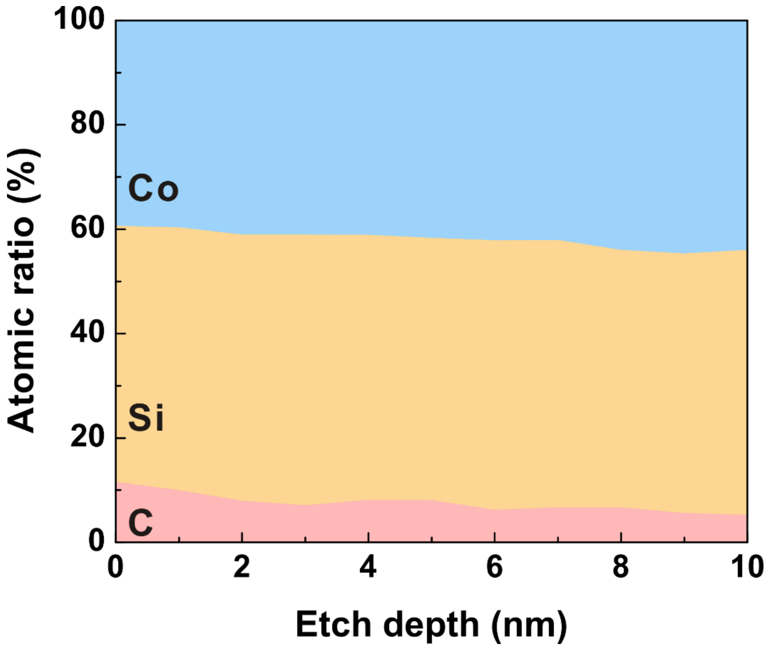

Supplementary Figure 35. Etching-XPS analysis of Co@$Si_{0.95}$ catalyst.

Note: The dispersion of Co and Si on the different depth of the sample was detected with etching-XPS technique. As shown in Supplementary Figure 35, the atomic ratio of Co and Si on Co@$Si_{0.95}$ catalyst has no obvious change by using carbon as an internal standard, suggesting the uniform dispersion of Co species on the sample.

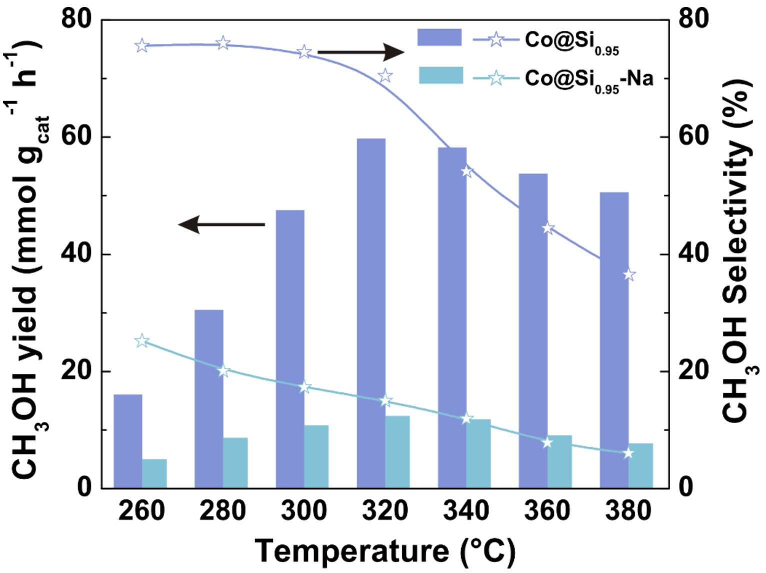

Supplementary Figure 36. Comparison of activity of Co@Si0.95 and NaOH-treated Co@Si0.95 (Co@Si0.95-Na) catalysts in CO_2 hydrogenation. Reaction conditions: 0.2 g of catalyst, 2.0 MPa, H_2/CO_2 = 3:1, GHSV = 6000 mL/g h.

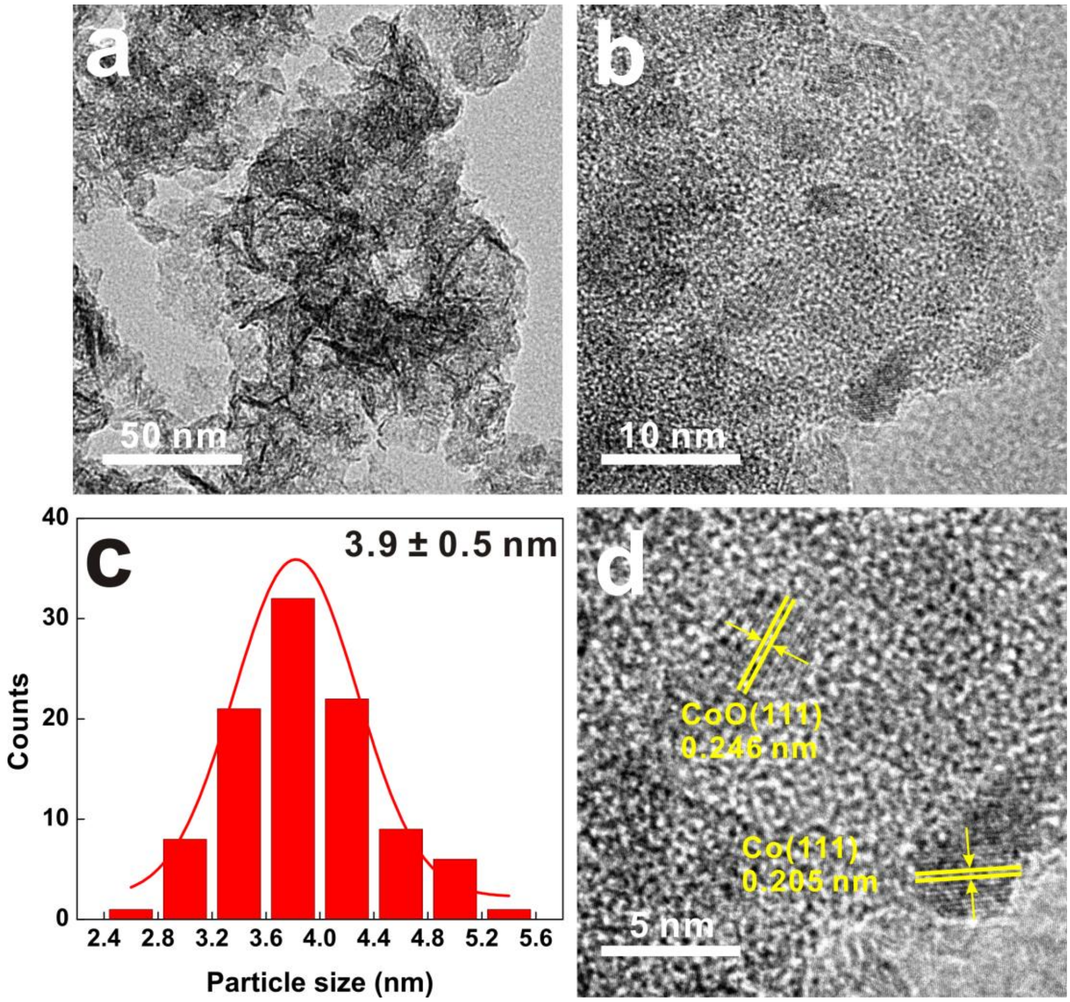

Supplementary Figure 37. (a, b) TEM images, (c) Co nanoparticle size distribution, and (d) HRTEM image of Co@$Si_{0.95}$ after CO_2 hydrogenation for 100 h. The reaction conditions are the same as those in Figure 2d in the main text.

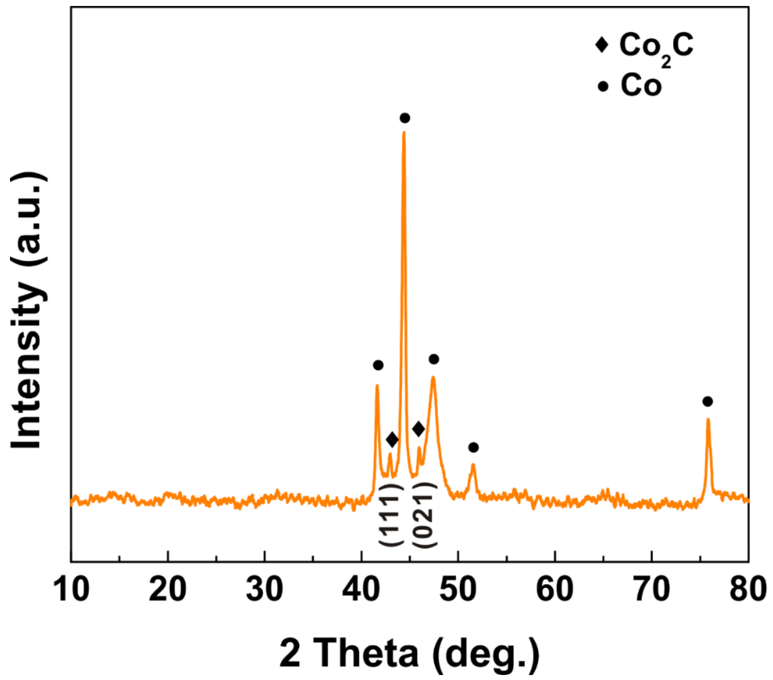

Supplementary Figure 38. XRD pattern of used Co/SiO_2 in CO_2 hydrogenation for 100 h. The reaction conditions are the same as those in Figure 2d in the main text.

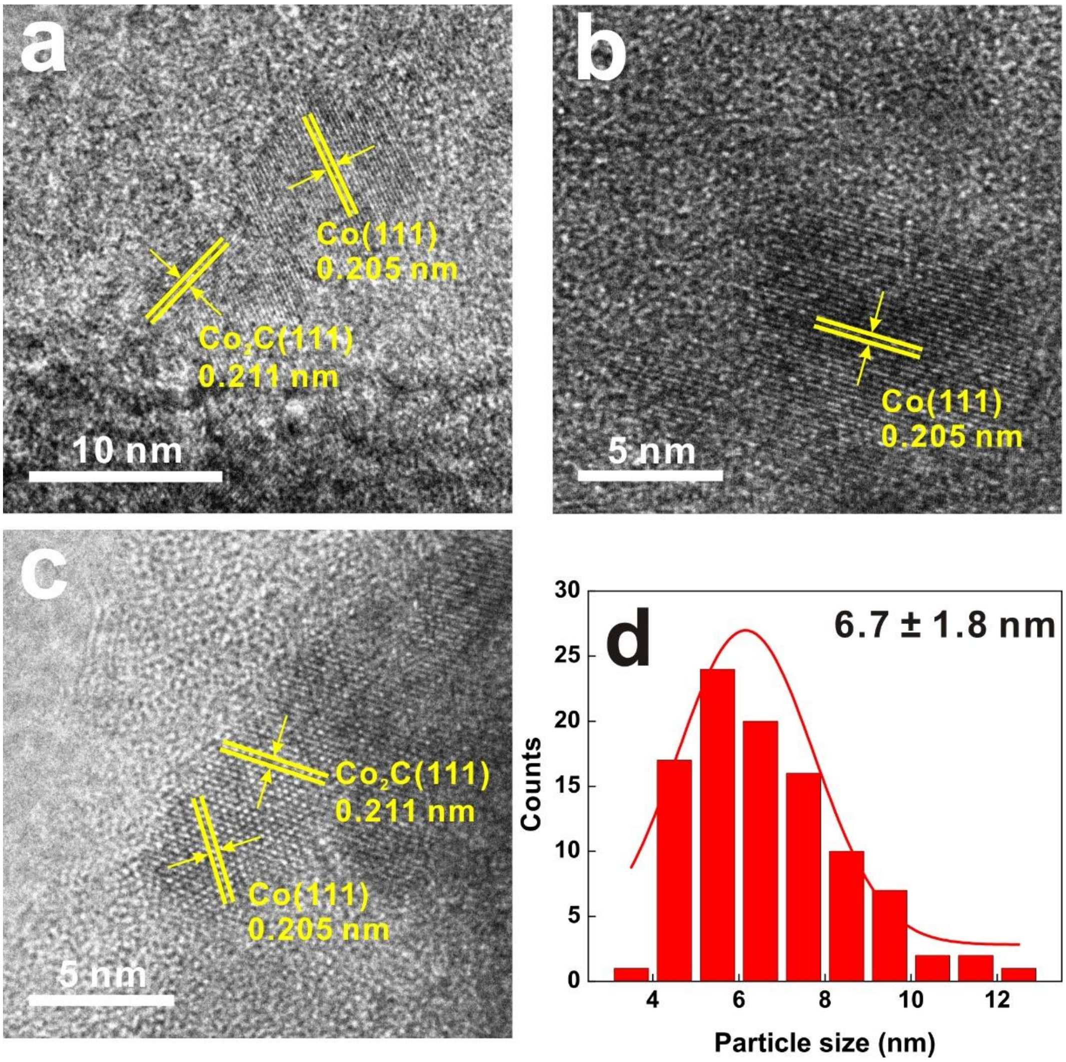

Supplementary Figure 39. (a–c) HRTEM images and (d) Co nanoparticle size distribution of Co/SiO_2 after CO_2 hydrogenation for 100 h. The reaction conditions are the same as those in Figure 2d in the main text.

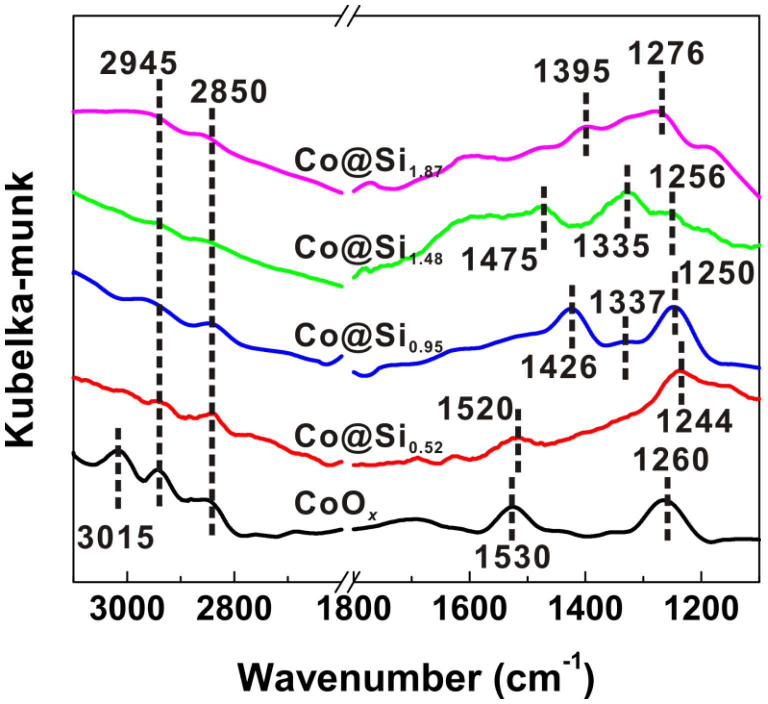

Supplementary Figure 40. *In-situ* DRIFTS spectra of various catalysts in flowing CO_2 at 250 °C.

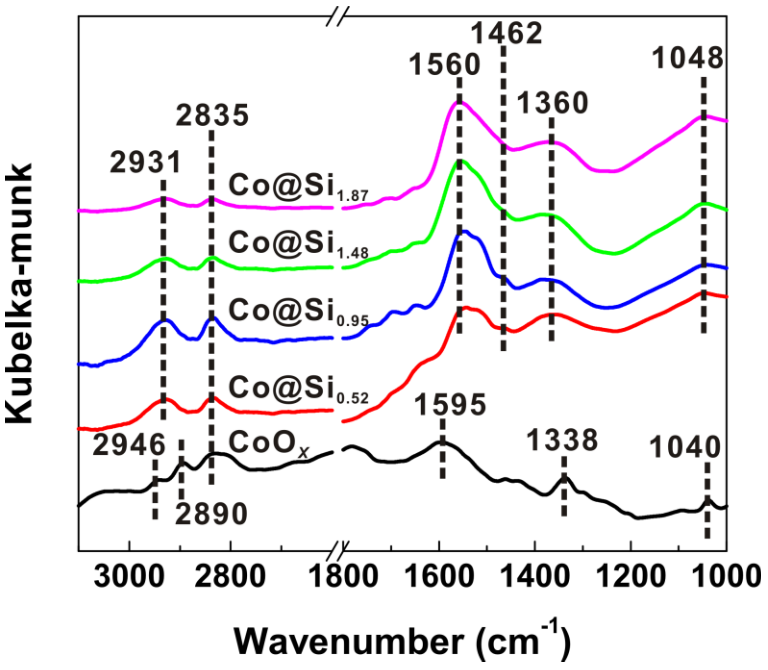

Supplementary Figure 41. *In-situ* DRIFTS spectra of Co@Si$_x$ catalysts at 350 °C with feed gases of CO_2 and H_2.

Note: CoO$_x$ gives DRIFTS bands at 1040, 1338, 1595, 2835, 2890 and 2946 cm^{-1}. The bands at 1040 and 2835 cm^{-1} are assigned to methoxy (*CH_3O) species, and band at 1595 cm^{-1} is attributed to $CO_2^{\delta-}$ species. The signals at 1338, 2890, and 2946 cm^{-1} are also observed assigned to the *HCOO species[21–23].

In contrast, the bands at 1048, 1360, 1462, 1560, 2835 and 2931 cm^{-1} are observed on the Co@Si$_x$ catalysts, indicating *CH_3O (1048, 1462, 2835 and 2931 cm^{-1}) and *HCOO (1360 and 1560 cm^{-1}) species are abundantly formed. Among all these catalysts, the Co@Si$_{0.95}$ exhibits the strongest *CH_3O signal, in agreement with its high methanol producibility in the CO_2 hydrogenation.

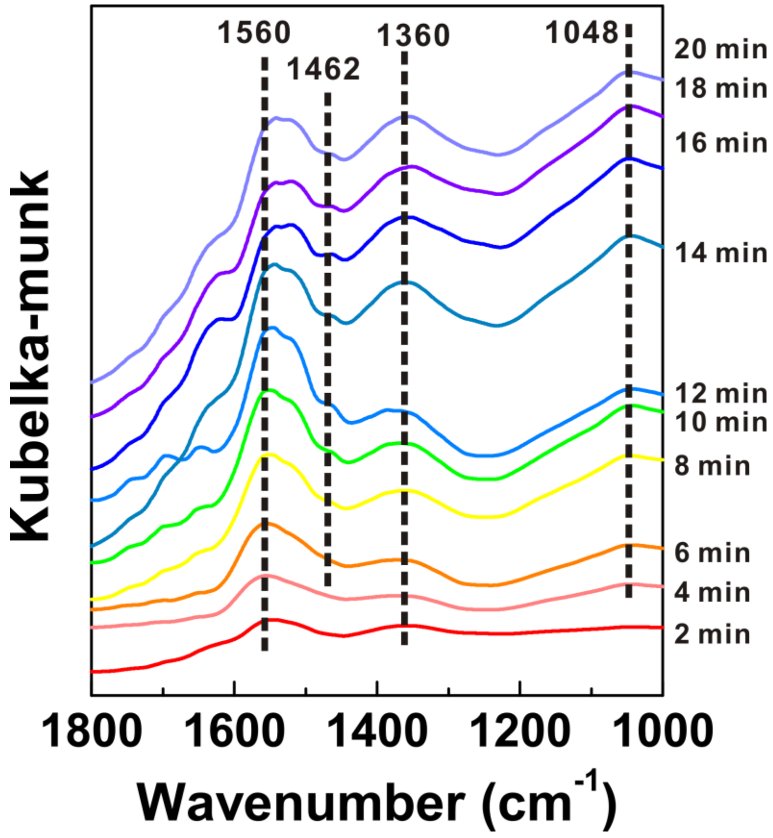

Supplementary Figure 42. *In-situ* DRIFTS spectra of CO_2 pretreated $Co@Si_{0.95}$ catalyst with pulsed H_2 at 350 °C.

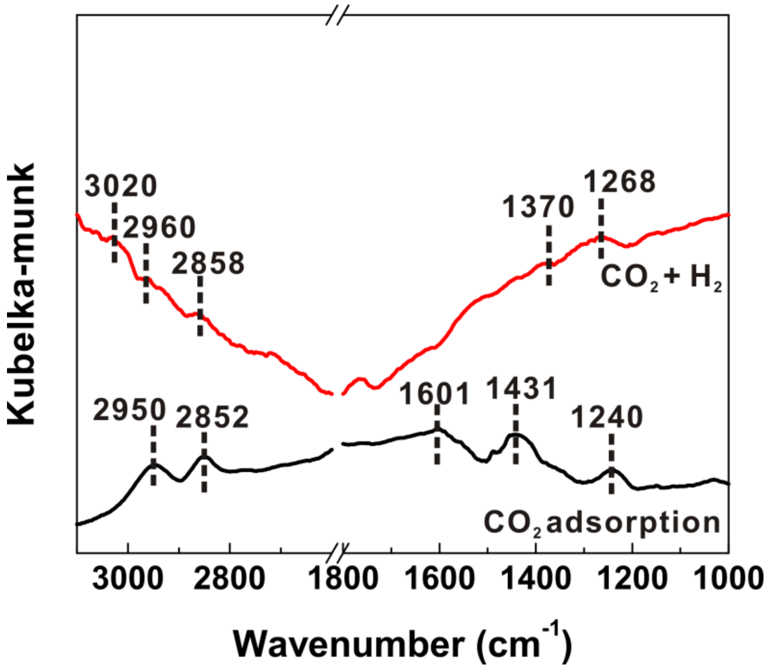

Supplementary Figure 43. *In-situ* DRIFTS spectra of Co/SiO_2 in CO_2 hydrogenation at 350 °C (feed gases of CO_2 and CO_2 + H_2).

Note: Co/SiO_2 gives DRIFTS bands at 1240, 1431, 1601, 2852 and 2950 cm^{-1}, assigned to the adsorbed $CO_2^{\delta-}$ (1240 and 1601cm^{-1}), CO_3^{2-} (1431 cm^{-1}) and *HCOO (2852 and 2950 cm^{-1}) in CO_2 atmosphere[21–23]. When the mixed gases of CO_2 and H_2 were introduced, new bands generated at 1268, 1370, 2858 and 3020 cm^{-1}, indicating the formation of more *HCOO (1370, 2858 and 2960 cm^{-1}) and *CH_x (3020 cm^{-1}) species[3].

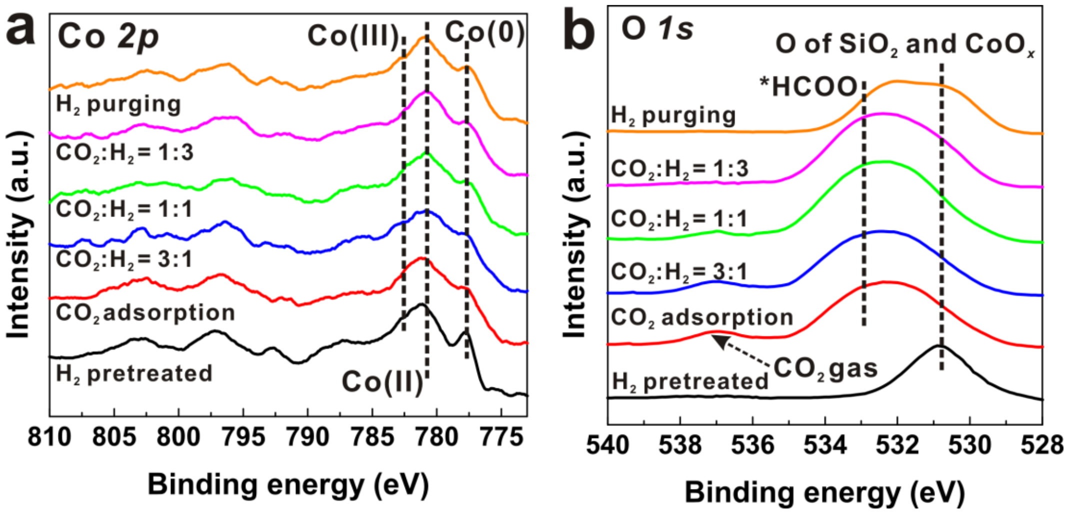

Supplementary Figure 44. *In-situ* (a) Co *2p* and (b) O *1s* XPS spectra of Co@$Si_{0.95}$ catalyst under 1.2 mbar CO_2 and H_2 atmosphere at 250 °C.

Note: The Co^0 signals in Co *2p* XPS spectra were weakened by introducing CO_2 to Co@$Si_{0.95}$ catalyst and reduced by feeding hydrogen, suggesting the adsorption and hydrogenation of CO_2 taking place on the Co sites. The signal of *HCOO appeared at 533.0 eV and was almost unchanged even under H_2 purging, confirming the highly stable *HCOO intermediate.

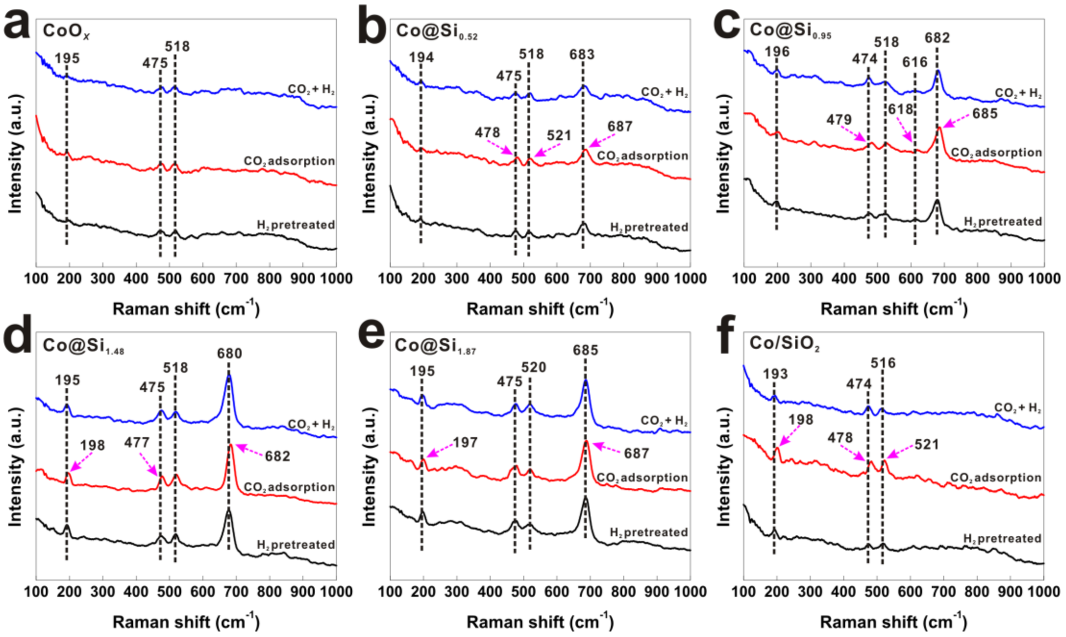

Supplementary Figure 45. *In-situ* Raman spectra of (a) CoO_x, (b–e) various Co@Si_x and (f) Co/SiO_2 catalysts under CO_2 and CO_2 + H_2 treatment at 250 °C.

Note: *In-situ* Raman spectra of Co@Si_x and Co/SiO_2 catalysts were collected to further investigate the change of cobalt in CO_2 adsorption and hydrogenation process. Co@$Si_{0.95}$ gives Raman peaks at 196, 474, 518, 616 and 682 cm^{-1}, assigned to the Co–O species. Introducing CO_2 gas to the Co@$Si_{0.95}$ sample slightly enhanced the peaks and resulted in the shift from 474, 616 and 682 cm^{-1} to 479, 618 and 685 cm^{-1}, respectively. This phenomenon might be due to the interaction of O on CO_2 with the cobalt surface. These peaks were reversed to the original position by introducing hydrogen, confirming the adsorbed CO_2 was transformed.

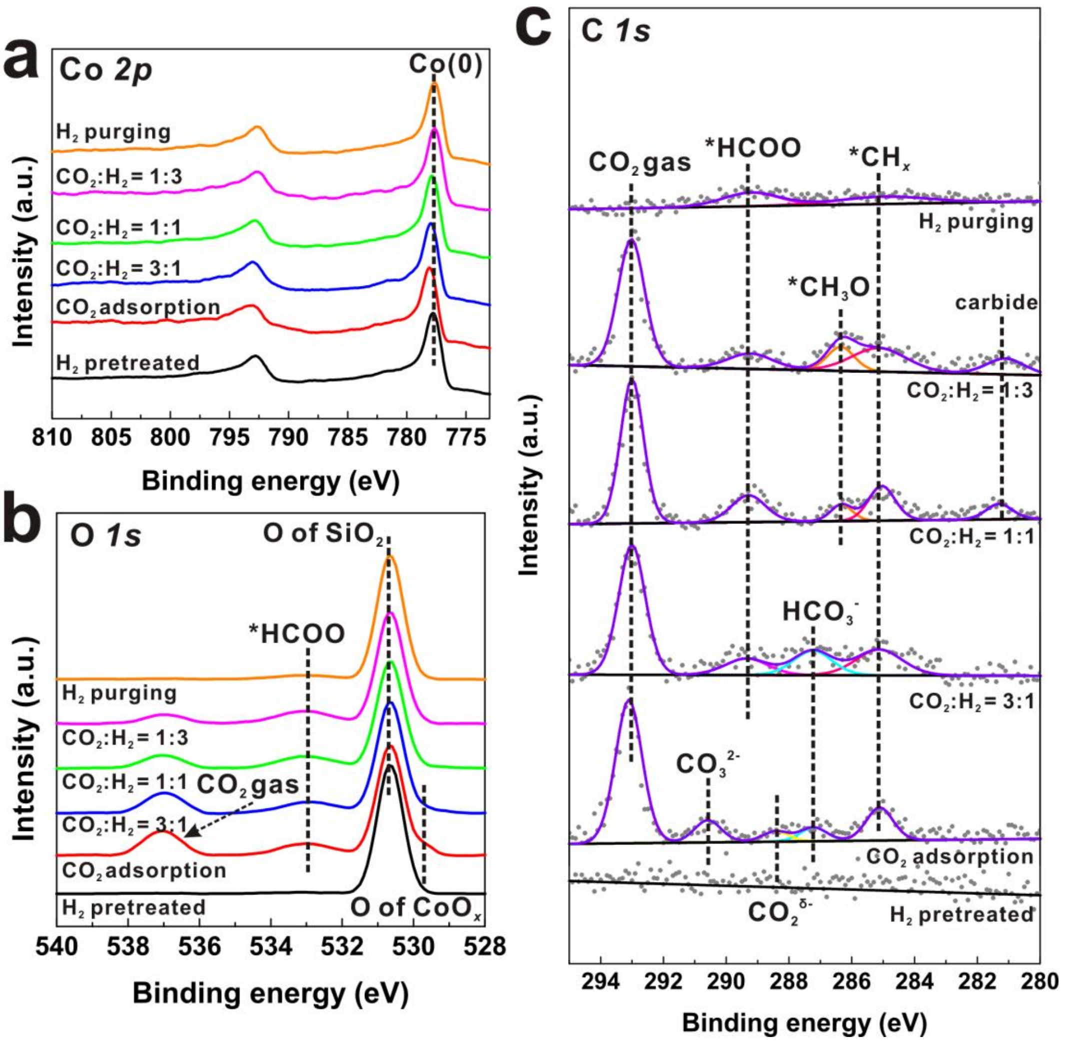

Supplementary Figure 46. *In-situ* (a) Co *2p*, (b) O *1s*, and (c) C *1s* XPS spectra of Co/SiO_2 under 1.2 mbar of mixed CO_2 and H_2 with different ratios at 250 °C.

Note: The CO_2 hydrogenation process was identified by *in-situ* XPS. The Co/SiO_2 sample gives peaks at 293.0, 290.6, 288.4, 287.2 and 285.1 eV in C *1s* XPS spectra after CO_2 adsorption treatment, assigned to gaseous CO_2, CO_3^{2-}, $CO_2^{\delta-}$, HCO_3^- and $*CH_x$ species, respectively[24]. When a slight amount of hydrogen was introduced over the sample (CO_2:H_2 at 3), signals of CO_3^{2-}, $CO_2^{\delta-}$, and HCO_3^- visibly changed and the *HCOO signal generated. Notably, a weak peak at 286.3 eV appeared by introducing more hydrogen, assigned to the $*CH_3O$ species. The $*CH_3O$ and $*CH_x$ signals were raised by continuously increasing the hydrogen content in the feed gas. A hydrogen purging treatment caused the elimination of almost all the peaks, because of their further hydrogenation and desorption from the catalyst surface. But the *HCOO signal still existed, confirming the highly stable *HCOO might not be the reaction intermediate.

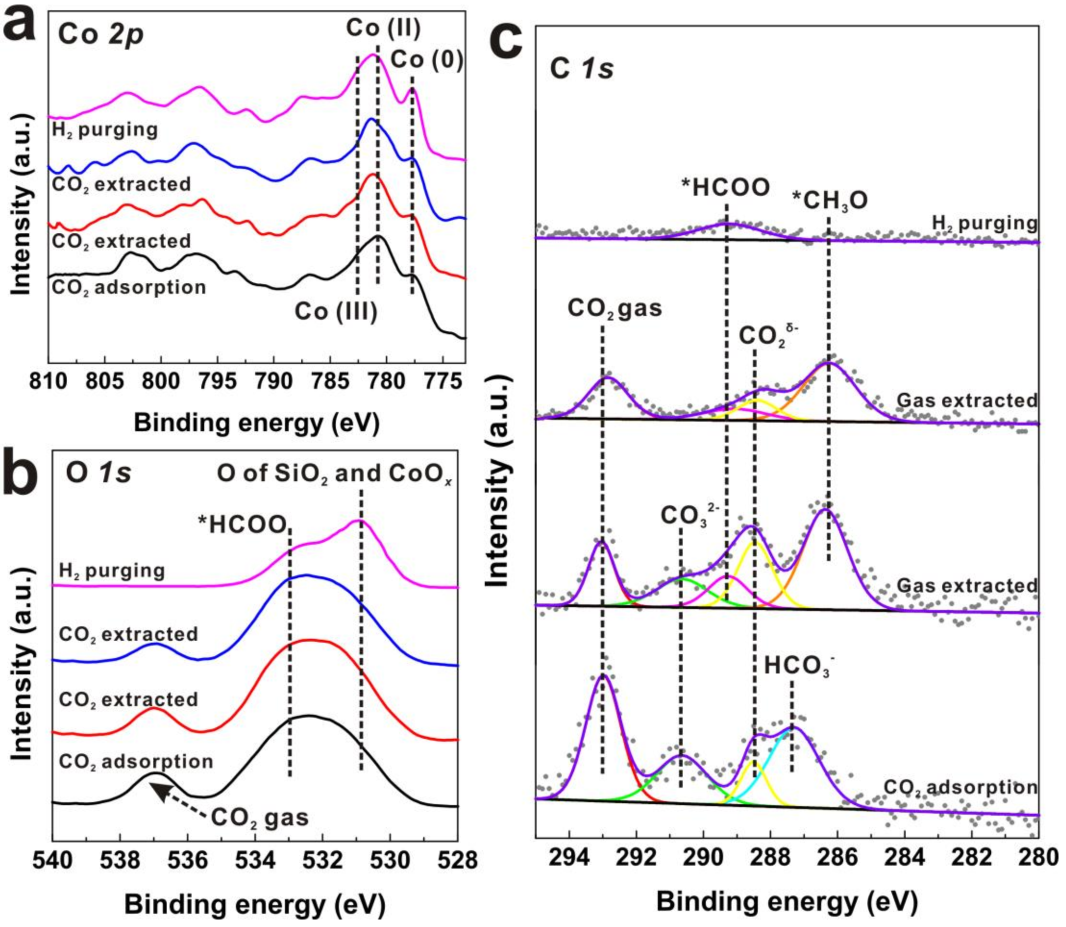

Supplementary Figure 47. *In-situ* (a) Co *2p*, (b) O *1s* and (c) C *1s* XPS spectra of Co@$Si_{0.95}$ under mixed gases (1.0 mbar CO_2 and 0.1 mbar H_2) treatment for 5 min, and slowly extracted from the chamber at 250 °C.

Note: The Co@$Si_{0.95}$ catalyst was further studied in the atmosphere of mixed gas with low hydrogen pressure (1.0 mbar CO_2 and 0.1 mbar H_2), giving C *1s* XPS peaks at 293.0, 290.6, 288.4 and 287.2 eV, assigned to gaseous CO_2, CO_3^{2-}, $CO_2^{\delta-}$ and HCO_3^- species, respectively (Supplementary Figure 47c). Then the CO_2 and H_2 gases were slowly extracted from the chamber, giving the strong signals at 289.2 and 286.3 eV, assigned to *HCOO and *CH_3O species, respectively. Although these signals were slightly reduced with the extracting treatment, because of their desorption or further transformation, they still exhibited obvious signals confirming their high stability on the catalyst surface. Finally, by purging with hydrogen, the *CH_3O peak immediately disappeared, suggesting its easy transformation in hydrogen, while the *HCOO was still stable, in good agreement with the abovementioned results.

The equivalent treatment was performed over the Co/SiO_2 catalyst (Supplementary Figure 48c), which gave the C *1s* XPS peaks at 293.0, 290.6, 287.2, and 285.1 eV assigned to gaseous CO_2, CO_3^{2-}, HCO_3^- and *CH_x species, respectively. When extracting the feed gases in the chamber, the *HCOO and *CH_x (289.2 and 285.1 eV) appeared dominantly with almost undetectable *CH_3O. In the end, the hydrogen

purging process eliminated other intermediates but *HCOO species was still unchanged. This phenomenon demonstrates the poor stability of *CH_3O species, which disappeared without further hydrogen introduction, suggesting its transformation might proceed the direct decomposition rather than direct hydrogenation (direct hydrogenation is a key step for methanol formation). This result is different from that on the Co@$Si_{0.95}$ catalyst, suggesting the important role of Co–O–SiO_n linkage for stabilizing the *CH_3O intermediate to avoid decomposition and benefiting the direct hydrogenation to form methanol.

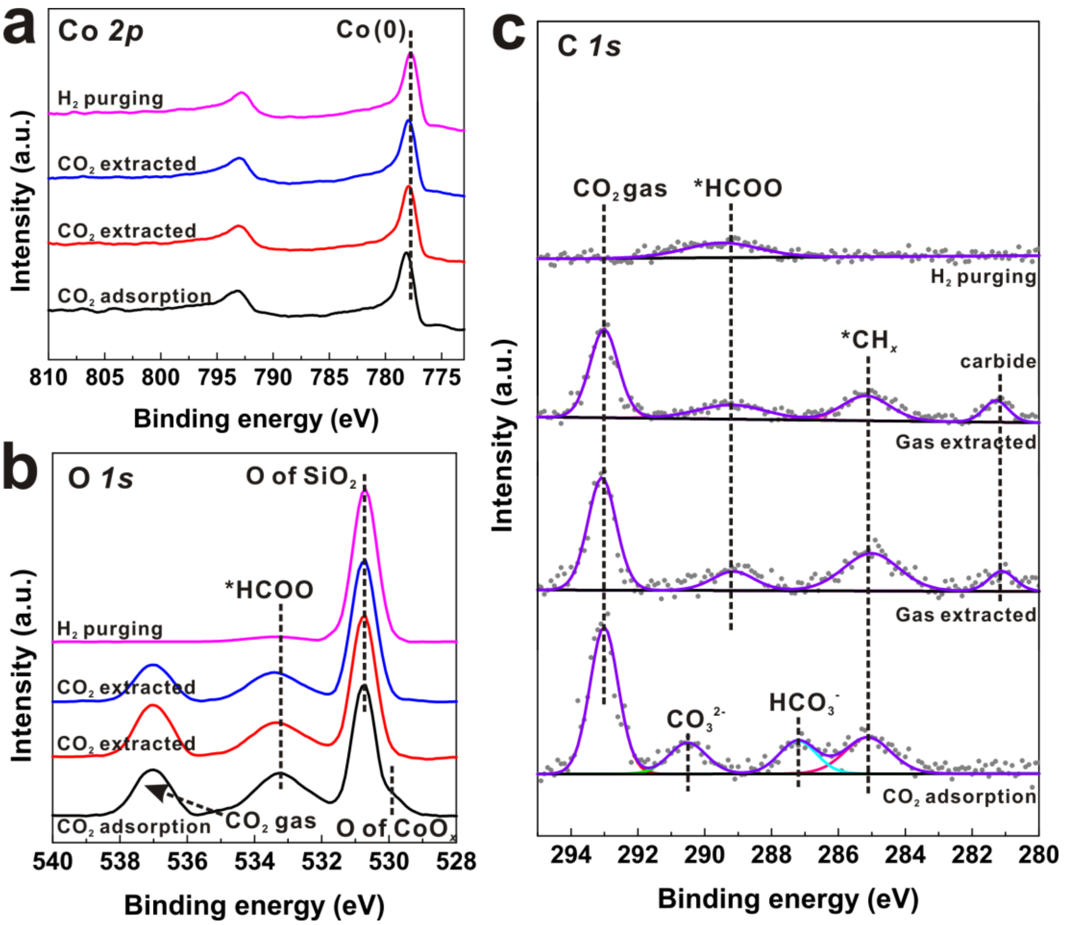

Supplementary Figure 48. *In-situ* (a) Co *2p*, (b) O *1s* and (c) C *1s* XPS spectra of Co/SiO_2 mixed gases (1.0 mbar CO_2 and 0.1 mbar H_2) treatment for 5 min, and slowly extracted from the chamber at 250 °C.

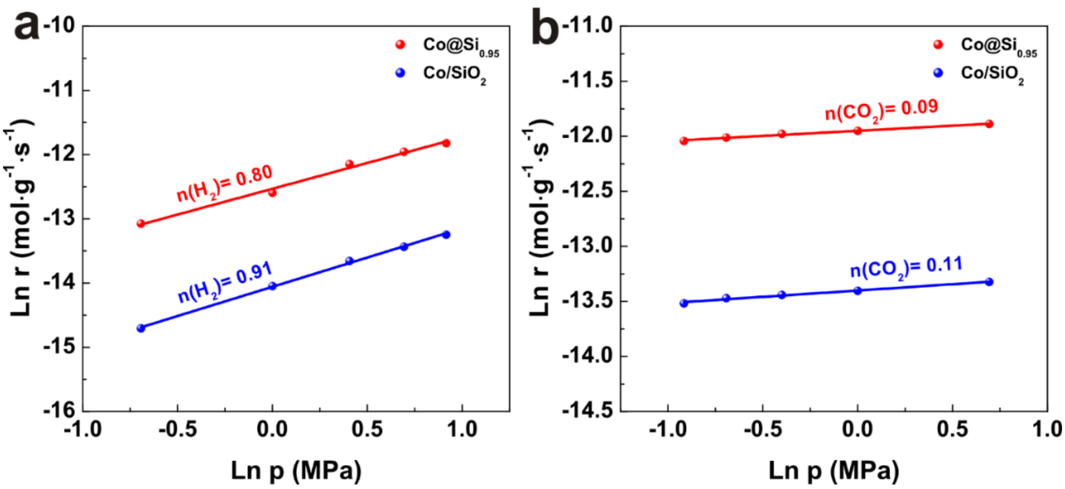

Supplementary Figure 49. The kinetic plots of Co@$Si_{0.95}$ and Co/SiO_2 in CO_2 hydrogenation on the basis of (a) H_2 and (b) CO_2 partial pressure.

Note: We performed pressure-dependent tests and showed the reaction orders of CO_2 and H_2. An approximately first-order dependence on the H_2 partial pressure is observed for methanol synthesis, giving 0.80 and 0.91 over Co@$Si_{0.95}$ and Co/SiO_2 catalysts, respectively. This result indicates that the H_2 positively influences the methanol formation in the CO_2 hydrogenation[25–31]. The apparent CO_2 reaction order in methanol synthesis is close to zero (0.09 for Co@$Si_{0.95}$ and 0.11 for Co/SiO_2), suggesting that the catalyst surface is saturated by CO_2 and/or the reaction intermediates, and higher hydrogen pressure could efficiently accelerate the whole reactions.

In the CO_2 hydrogenation to methanol, it is generally recognized that there are two major reaction pathways based on experimental observation and theoretical calculations, including the RWGS + CO hydrogenation pathway and the formate pathway[1,23,25,32–38]. Different rate-control steps have been suggested for methanol pathway from CO_2 and H_2 *via* these pathways. For example, in the formate pathway, hydrogenation of *HCOO (*HCOO + H → *HCOOH) and *CH_3O (*CH_3O + H → *CH_3OH) intermediates are the rate-control steps[1,23,32]. As reported previously, if the hydrogenation of *HCOO is the rate-control step, the maximum attainable reaction orders according are 1 for both CO_2 and H_2. Generally, H* and HCOO* are always abundant on the catalyst surface[25,26,29], the apparent CO_2 and H_2 reaction orders would be much smaller than 1, which is inconsistent with our results (H_2 reaction order is close to 1). These data confirm that the H_2 or CO_2 activation should not be the control step. Our *in-situ* DRIFTS and XPS results demonstrate that the *CH_3O was abundantly detected on the catalyst surface in CO_2 and H_2 atmosphere, and the further transformation of *CH_3O was slow, suggesting the *CH_3O hydrogenation should be the rate-control step. The low reaction order of CO_2 (0.09–0.11) can be attributed to the slow step of *CH_3O hydrogenation to CH_3OH as a rate-control step[25,26]. Similarly, if CO_2 hydrogenation follows the RWGS + CO hydrogenation pathway, the *CH_3O is also the crucial intermediate for CH_3OH formation, and *CH_3O hydrogenation to CH_3OH is the rate-control step[1,23,32]. These kinetic data demonstrate that methanol

production over $Co@Si_{0.95}$ and Co/SiO_2 catalysts follows similar route that $*CH_3O$ hydrogenation to CH_3OH is the rate-control step.

The apparent E_a for methane and CO production on $Co@Si_{0.95}$ are 135.5 and 53.2 kJ mol^{-1}, respectively. These values are remarkably higher than those over the Co/SiO_2 catalyst (96.4 kJ mol^{-1} for methane and 43.1 kJ mol^{-1} for CO production). These data confirm that easier methane and CO formation on Co/SiO_2 catalyst compared with $Co@Si_{0.95}$. In addition, the $Co@Si_{0.95}$ exhibited apparent E_a for methanol production at 58.2 kJ mol^{-1}, lower than 62.4 kJ mol^{-1} on Co/SiO_2 catalyst, which suggests easier methanol production on $Co@Si_{0.95}$ catalyst.

The $Co@Si_{0.95}$ catalyst exhibits slightly higher CO_2 conversion than that of traditional Co/SiO_2 or CoO_x catalysts. Although the former is unfavorable for H_2 dissociation because of the lower metallic Co content. Actually, similar phenomenon has been observed previously in different reaction systems, where the catalysts with lower ability for reactant activation might exhibit faster transformation rate[39–42]. This phenomenon is explained by the balanced rates for the production and further transformation of key intermediates, which have been studied in various reaction systems previously[41–45].

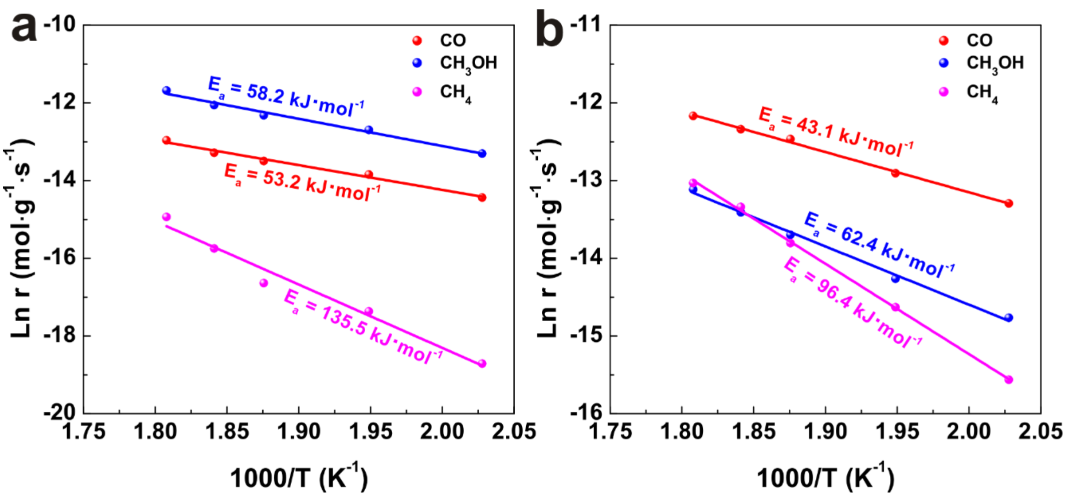

Supplementary Figure 50. Arrhenius plots for CO, CH_3OH, and CH_4 formation over (a) Co@$Si_{0.95}$ and (b) Co/SiO_2 catalysts.

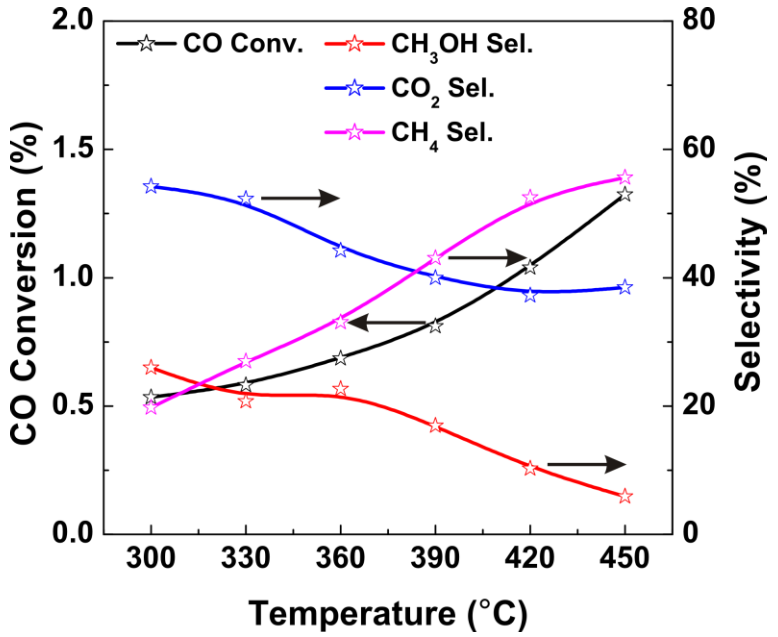

Supplementary Figure 51. Dependences of the CO conversion, products selectivity of the Co@Si$_{0.95}$ catalyst for CO hydrogenation. Reaction conditions: 0.2 g of catalyst, 2.0 MPa, H_2/CO = 2:1, GHSV = 6000 mL/g h.

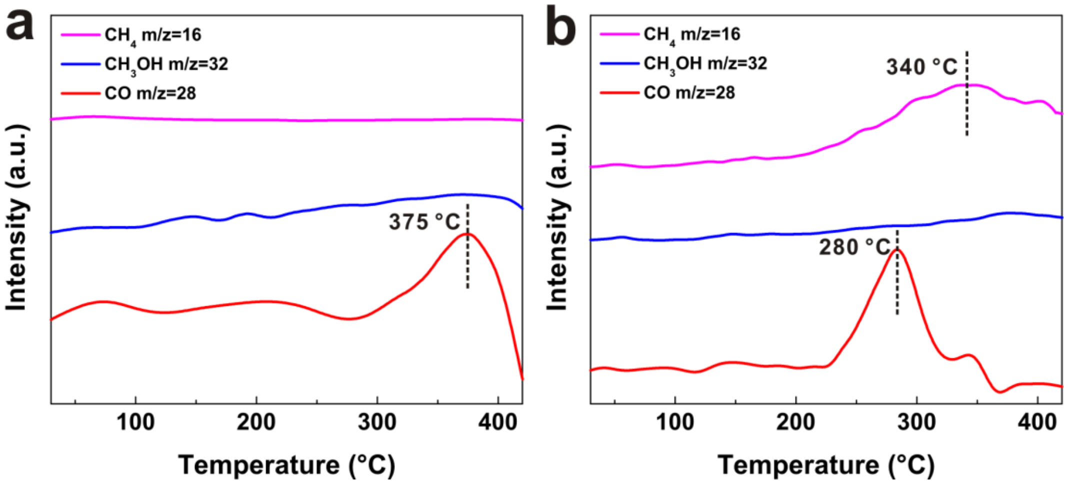

Supplementary Figure 52. MeOH-TPSR profiles of (a) Co@$Si_{0.95}$ and (b) Co/SiO_2 catalysts.

Note: CO is usually formed in the cobalt-catalyzed CO_2 hydrogenation reactions. With regard to CO formation, it is recognized that there are two major reaction pathways for the CO_2-to-CH_3OH transformation based on experimental observation and theoretical calculations, including the RWGS + CO hydrogenation pathway and the formate pathway[1,23,25,32–38]. In the RWGS + CO hydrogenation pathway, the *CO intermediate is firstly produced from the RWGS reaction *via* the *HOCO intermediate, or the direct C–O bond cleavage of *CO_2. The *CO intermediate is further hydrogenated to *CH_3O, which could be transformed into CH_3OH. Simultaneously, the *CO species could also desorb from the catalyst directly to form CO, which have been reported on the Co-based catalysts[2,4,5,7,10]. The formate pathway proceeds the *HCOO species by the primary hydrogenation of CO_2, which produces *CH_3O *via* the C–O bond cleavage of the *H_xCOOH intermediates, and is eventually hydrogenated to the CH_3OH. According to such proposed pathway, it seems the reaction does not produce CO[1]. However, we cannot exclude the CO production from the formate route because the formic acid is easily decomposed into CO and water on the metal catalysts[46–48]. In addition, the *CH_3O and methanol species could be decomposed into CO species.

Supplementary Table 1. Atomic ratio of Si/Co on several Co@Si_x catalysts.

Entry	Sample	Si/Co (molar ratio) by ICP analysis
1	CoO_x	0.01
2	Co@$Si_{0.52}$	0.52
3	Co@$Si_{0.95}$	0.95
4	Co@$Si_{1.48}$	1.48
5	Co@$Si_{1.87}$	1.87
6	Co@$Si_{0.95}$[a]	0.97
7	Co/SiO_2	1.29

[a]The sample was obtained after CO_2 hydrogenation over 100 h.

Supplementary Table 2. The surface area and volume data of various samples.

Entry	Sample	S_{BET} (m^2/g)	V_{meso} (cm^3/g)
1	CoO_x	59.7	0.16
2	$Co@Si_{0.52}$	151.3	0.22
3	$Co@Si_{0.95}$	147.4	0.17
4	$Co@Si_{1.48}$	134.6	0.18
5	$Co@Si_{1.87}$	157.6	0.20
6	SiO_n	111.4	0.15
7	Co/SiO_2	123.5	0.26

S_{BET}: BET specific surface area; V_{meso}: mesopore volume.

Note: As shown in Supplementary Table 2, the $Co@Si_x$ and Co/SiO_2 catalysts showed the surface areas at 123.5–157.6 m^2/g and mesoporous volumes at 0.17–0.26 cm^3/g. The similar mesoporosity demonstrates that the comparable mass transfer on these catalysts, which should not be the reason for the different catalytic performances.

Supplementary Table 3. Comparison of the activities of various catalysts for the CO_2 hydrogenation to methanol.

Catalyst	Temp. (°C)	Press. (MPa)	CO_2 Conv. (%)	Methanol sel. (%)	Methanol productivity (mmol/g_{cat}·h)	Ref.
**Co@$Si_{0.95}$**	320	2	8.6	70.5	59.7	This work
**Cu/ZrO_2**	220	0.1	0.27	28.3	0.93	23
**Cu/TiO_2**	220	0.1	0.26	19.1	0.04	23
**Cu/ZrO_2**	230	2.5	–[a]	75.0	0.43	49
**Cu/SiO_2**	230	2.5	–	49.0	0.07	49
**Cu/m-ZrO_2**	230	1	3.8	67.0	1.4	50
**Pd–Cu–ZnO**	270	4.5	8.0	64.0	4.6	51
**Cu/ZnO**	230	3	–	–	9.7	52
**$PdCu_3$**	270	5	2.8	18.5	1.6	53
**Cu/SiO_2**	320	3	28.1	21.3	5.7	54
**CuZnGa**	270	4.5	19.5	48.0	12.8	55
**Ni–Ga**	200	0.1	–	–	2.0	56
**In_2O_3/ZrO_2**	300	5	5.2	99.8	6.4	57
**ZrZnCu**	220	8	–	–	3.5	58
**CuZnZrGaY**	240	2	–	–	13.8	59
**Pd_2Ga**	210	5	–	64.0	35.0	60
**Cu/ZnO/Al_2O_3**	230	6	–	–	42.0	61
**Ni–In–Al/SiO_2**	260	0.1	2.8	2.3	0.59	62
**Cu/Zr@SiO_2**	230	2.5	–	77.0	1.0	63
**Pd/ZnO**	250	2	10.7	60.0	2.4	64
**Pt_1@MIL**	200	3.2	–	~90	7.2	65
**7.5% Pt/MoS_2**	210	3.2	–	76.0	12.2	66

[a] Not shown in the references.

Supplementary Table 4. EXAFS structure parameters representing Co@Si$_x$ and Co/SiO$_2$ samples[a].

Sample	*N* (Co–O)	*R* (Co–O) (Å)	*N* (Co–Co)	*R* (Co–Co) (Å)	σ^2 (Co–O) (Å^2)	σ^2 (Co–Co) (Å^2)
**Co/SiO$_2$**	–	–	9.3	2.50	–	0.054
**Co@Si$_{0.52}$**	3.8	2.05	10.7	3.02	0.008	0.014
**Co@Si$_{0.95}$**	4.2	2.05	10.7	3.02	0.008	0.014
**Co@Si$_{1.87}$**	4.5	2.05	6.3	3.07	0.009	0.019

[a]*N*, coordination number; *R*, distance between absorber and backscatterer atoms; σ^2, disorder term (EXAFS Debye–Waller factor). Amplitude factor S_0^2 = 0.83. For Co/SiO$_2$, *k*-range: 3.0–14.0 Å^{-1}, *R*-range: 1.0–3.0 Å, E_0 = 9.26 ± 0.21 eV; for Co@Si$_x$, *k*-range: 3.0–13.9 Å^{-1}, *R*-range: 1.0–3.2 Å, E_0 = -3.01 ± 0.33 eV. Estimated EXAFS error bounds: *N*, ±20%; *R*, ±0.01 Å; $\Delta\sigma^2$, ±20%.

References

1. Kattel, S., Liu, P. & Chen, J. G. Tuning selectivity of CO_2 hydrogenation reactions at the metal/oxide interface. *J. Am. Chem. Soc.* **139**, 9739–9754 (2017).
2. Porosoff, M. D., Yang, X., Boscoboinik, J. A. & Chen, J. G. Molybdenum carbide as alternative catalysts to precious metals for highly selective reduction of CO_2 to CO. *Angew. Chem. Int. Ed.* **53**, 6705–6709 (2014).
3. Kattel, S., Yu, W., Yang, X., Yan, B., Huang, Y., Wan, W., Liu, P. & Chen, J. G. CO_2 Hydrogenation over oxide-supported PtCo catalysts: The role of the oxide support in determining the product selectivity. *Angew. Chem. Int. Ed.* **55**, 7968–7973 (2016).
4. Ilsemann, J., Straß-Eifert, A., Friedland, J., Kiewidt, L., Thöming, J., Bäumer, M. & Güttel, R. Cobalt@silica core-shell catalysts for hydrogenation of CO/CO_2 mixtures to methane. *ChemCatChem* **11**, 4884–4893 (2019).
5. Schubert, M., Pokhrel, S., Thomé, A., Zielasek, V., Gesing, T. M., Roessner, F., Mädler, L. & Bäumer, M. Highly active Co–Al_2O_3-based catalysts for CO_2 methanation with very low platinum promotion prepared by double flame spray pyrolysis. *Catal. Sci. Technol.* **6**, 7449–7460 (2016).
6. Kiezkowska-Pawlak, H., Tyczkowski, J., Balcerzak, J. & Tracz, P. Advances in plasma produced CoO_x-based nanocatalysts for CO_2 methanation. *Catal. Today* **337**, 162–170 (2019).
7. Li, W., Zhang, G., Jiang, X., Liu, Y., Zhu, J., Ding, F., Liu, Z., Guo, X. & Song, C. CO_2 hydrogenation on unpromoted and M-promoted Co/TiO_2 catalysts (M = Zr, K, Cs): Effects of crystal phase of supports and metal–support interaction on tuning product distribution. *ACS Catal.* **9**, 2739−2751 (2019).
8. Li, W., Nie, X., Jiang, X., Zhang, A., Ding, F., Liu, M., Liu, Z., Guo, X. & Song, C. ZrO_2 support imparts superior activity and stability of Co catalysts for CO_2 methanation. *Appl. Catal. B* **220**, 397–408 (2018).
9. Zhou, G., Liu, H., Xing, Y., Xu, S., Xie, H. & Xiong, K. CO_2 hydrogenation to methane over mesoporous Co/SiO_2 catalysts: Effect of structure. *J. CO_2 Util.* **26**, 221–229 (2018).
10. Shin, H. H., Lu, L., Yang, Z., Kiely, C. J. & Mclntosh, S. Cobalt catalysts decorated with platinum atoms supported on barium zirconate provide enhanced activity and selectivity for CO_2 methanation. *ACS Catal.* **6**, 2811−2818 (2016).
11. Kiezkowska-Pawlak, H., Tracz, P., Redzynia, W. & Tyczkowski, J. Plasma deposited novel nanocatalysts for CO_2 hydrogenation to methane. *J. CO_2 Util.* **17**, 312–319 (2017).
12. Yin, X. J., Peng, K., Hu, A. P., Zhou, L. P., Chen, J. H. & Du, Y. W. Preparation and characterization of core–shell structured Co/SiO_2 nanosphere. *J. Alloy. Compd.* **479**, 372–375 (2009).
13. Huang, X. & Chen, Z. Preparation of $CoFe_2O_4$/SiO_2 nanocomposites by sol–gel method. *J. Cryst. Growth* **271**, 287–293 (2004).
14. Saib, A. M., Borgna, A., van de Loosdrecht, H., van Berge, P. J. & Niemantsverdriet, J. W. XANES study of the susceptibility of nano-sized cobalt crystallites to oxidation during realistic Fischer–Tropsch synthesis. *Appl. Catal. A* **312**, 12–19 (2006).
15. Wang, J., Zhou, J., Hu, Y. & Regier, T. Chemical interaction and imaging of single Co_3O_4/graphene sheets studied by scanning transmission X-ray microscopy and X-ray absorption spectroscopy. *Energy Environ. Sci.* **6**, 926–934 (2013).
16. Jiang, J. & Li, L. Synthesis of sphere-like Co_3O_4 nanocrystals via a simple polyol route. *Mater.*

Lett. **61**, 4894–4896 (2007).

17. Yang, J., Liu, H., Martens, W. N. & Frost, R. L. Synthesis and characterization of cobalt hydroxide, cobalt oxyhydroxide, and cobalt oxide nanodiscs. *J. Phys. Chem. C* **114**, 111–119 (2010).
18. Gong, J., Yue, H., Zhao, S., Zhao, L., Lv, J., Wang, S. & Ma, X. Synthesis of ethanol via syngas on Cu/SiO_2 catalysts with balanced Cu^0–Cu^+ sites. *J. Am. Chem. Soc.* **134**, 13922–13925 (2012).
19. Yue, H., Zhao, Y., Zhao, S., Wang, B., Ma, X. & Gong, J. A copper-phyllosilicate core-sheath nanoreactor for carbon–oxygen hydrogenolysis reactions. *Nat. Commun.* **4**, 2339 (2013).
20. Xu, C., Chen, G., Zhao, Y., Liu, P. Duan, X., Gu, L., Fu, G., Yuan, Y. & Zheng, N. Interfacing with silica boosts the catalysis of copper. *Nat. Commun.* **9**, 3367 (2018).
21. Kim, Y., Trung, T. S. B., Yang, S., Kim, S. & Lee, H. Mechanism of the surface hydrogen induced conversion of CO_2 to methanol at Cu(111) step sites. *ACS Catal.* **6**, 1037–1044 (2016).
22. Fisher, I. A. & Bell, A. T. *In-situ* infrared study of methanol synthesis from H_2/CO_2 over Cu/SiO_2 and Cu/ZrO_2/SiO_2 *J. Catal.* **172**, 222–237 (1997).
23. Kattel, S., Yan, B., Yang, Y., Chen, J. G. & Liu, P. Optimizing binding energies of key intermediates for CO_2 hydrogenation to methanol over oxide-supported copper. *J. Am. Chem. Soc.* **138**, 12440–12450 (2016).
24. Mudiyanselage, K., Senanayake, S. D., Feria, L., Kundu, S., Baber, A. E., Graciani, J., Vidal, A. B., Agnoli, S., Evans, J., Chang, R., Axnanda, S., Liu, Z., Sanz, J. F., Liu, P., Rodriguez, J. A. & Stacchiola, D. J. Importance of the metal–oxide interface in catalysis: *In situ* studies of the water–gas shift reaction by ambient-pressure X-ray photoelectron spectroscopy. *Angew. Chem. Int. Ed.* **52**, 5101–5105 (2013).
25. Grabow, L. C. & Mavrikakis, M. Mechanism of methanol synthesis on Cu through CO_2 and CO hydrogenation. *ACS Catal.* **1**, 365–384 (2011).
26. Medina, J. C., Figueroa, M., Manrique, R., Pereira, J. R., Srinivasan, P. D., Bravo-Suárez, J. J., Medrano, V. G. B., Jiménez, R., & Karelovic, A. Catalytic consequences of Ga promotion on Cu for CO_2 hydrogenation to methanol. *Catal. Sci. Technol.* **7**, 3375–3387 (2017).
27. Chen, T. Y., Cao, C., Chen, T. B., Ding, X., Huang, H., Shen, L., Cao, X., Zhu, M., Xu, J., Gao, J. & Han, Y. F. Unraveling highly tunable selectivity in CO_2 hydrogenation over bimetallic In–Zr oxide catalysts. *ACS Catal.* **9**, 8785−8797 (2019).
28. Karelovic, A., Galdames, G., Medina, J. C., Yévenes, C., Barra, Y. & Jiménez, R. Mechanism and structure sensitivity of methanol synthesis from CO_2 over SiO_2-supported Cu nanoparticles. *J. Catal.* **369**, 415–426 (2019).
29. Askgaard, T. S., Norskov, J. K., Ovesen, C. V. & Stoltze, P. A kinetic model of methanol synthesis. *J. Catal.* **156**, 229–242 (1995).
30. Arena, F., Mezzatesta, G., Zafarana, G., Trunfio, G., Frusteri, F. & Spadaro, L. Effects of oxide carriers on surface functionality and process performance of the Cu–ZnO system in the synthesis of methanol via CO_2 hydrogenation. *J. Catal.* **300**, 141–151 (2013).
31. Kobl, K., Thomas, S., Zimmermann, Y., Parkhomenko, K. & Roger, A. Power-law kinetics of methanol synthesis from carbon dioxide and hydrogen on copper–zinc oxide catalysts with alumina or zirconia supports. *Catal. Today* **270**, 31–42 (2016).
32. Kattel, S., Ramírez, P. J., Chen, J. G., Rodriguez, J. A. & Liu, P. Active sites for CO_2 hydrogenation to methanol on Cu/ZnO catalysts. *Science* **355**, 1296–1299 (2017).

33. Behrens, M., Studt, F., Kasatkin, I., Kühl, S., Hävecker, M., Abild-Pedersen, F., Zander, S., Girgsdies, F., Kurr, P., Kniep, B., Tovar, M., Fischer, R. W., Nørskov, J. K. & Schlögl, R. The active site of methanol synthesis over Cu/ZnO/Al_2O_3 industrial catalysts. *Science* **336**, 893–897 (2012).
34. Graciani, J., Mudiyanselage, K., Xu, F., Baber, A. E., Evans, J., Senanayake, S. D., Stacchiola, D. J., Liu, P., Hrbek, J., Sanz, J. F. & Rodriguez, J. A. Highly active copper–ceria and copper–ceria–titania catalysts for methanol synthesis from CO_2. *Science* **345**, 546–550 (2014).
35. Liu, C., Yang, B., Tyo, E., Seifert, S., DeBartolo, J., von Issendorff, B., Zapol, P., Vajda, S. & Curtiss, L. A. Carbon dioxide conversion to methanol over size-selected Cu_4 clusters at low pressures. *J. Am. Chem. Soc.* **137**, 8676−8679 (2015).
36. Tang, Q. L., Hong Q. J. & Liu, Z. P. CO_2 fixation into methanol at Cu/ZrO_2 interface from first principles kinetic Monte Carlo. *J. Catal.* **263**, 114−122 (2009).
37. Ye, J., Liu, C., Mei, D. & Ge, Q. Active oxygen vacancy site for methanol synthesis from CO_2 hydrogenation on In_2O_3(110): A DFT study. *ACS Catal.* **3**, 1296−1306 (2013).
38. Studt, F., Behrens, M., Kunkes, E. L., Thomas, N., Zander, S., Tarasov, A., Schumann, J., Frei, E., Varley, J. B., Abild-Pedersen, F., Nørskov, J. K. & Schlögl, R. *ChemCatChem* **7**, 1105−1111 (2015).
39. Pan, X., Fan, Z., Chen, W., Ding, Y., Luo, H. & Bao, X. Enhanced ethanol production inside carbon-nanotube reactors containing catalytic particles. *Nat. Mater.* **6**, 507–511 (2007).
40. Yang, G., Tsubaki, N., Shamoto, J., Yoneyama, Y. & Zhang, Y. Confinement effect and synergistic function of H-ZSM-5/Cu-ZnO-Al_2O_3 capsule catalyst for one-step controlled synthesis. *J. Am. Chem. Soc.* **132**, 8129–8136 (2010).
41. Xiao, J., Pan, X., Guo, S., Ren, P. & Bao, X. Toward fundamentals of confined catalysis in carbon nanotubes. *J. Am. Chem. Soc.* **137**, 477–482 (2015).
42. Wang, C., Wang, L., Zhang, J., Wang, H., Lewis, J. P. & Xiao, F.-S. Product selectivity controlled by zeolite crystals in biomass hydrogenation over a palladium catalyst. *J. Am. Chem. Soc.* **138**, 7880–7883 (2016).
43. Yang, N., Yoo, J. S., Schumann, J., Bothra, P., Singh, J. A., Valle, E., Abild-Pedersen, F., Nørskov, J. K. & Bent, S. F. Rh–MnO interface sites formed by atomic layer deposition promote syngas conversion to higher oxygenates. *ACS Catal.* **7**, 5746-5757 (2017)
44. Cao, A., Schumann, J., Wang, T., Zhang, L., Xiao, J., Bothra, P., Liu, Y., Abild-Pedersen, F. & Nørskov, J. K. Mechanistic insights into the synthesis of higher alcohols from syngas on CuCo alloys. *ACS Catal.* **8**, 10148–10155 (2018).
45. Huang, X., Teschner, D., Dimitrakopoulou, M., Fedorov, A., Frank, B., Kraehnert, R., Rosowski, F., Kaiser, H., Schunk, S., Kuretschka, C., Schlögl, R., Willinger, M. & Trunschke, A. Atomic-scale observation of the metal–promoter interaction in Rh-based syngas-upgrading catalysts. *Angew. Chem. Int. Ed.* **58**, 8709–8713 (2019).
46. Wang, Z. L., Yan, J. M., Ping, Y., Wang, H. L., Zheng, W. T. & Jiang, Q. An efficient CoAuPd/C catalyst for hydrogen generation from formic acid at room temperature. *Angew. Chem. Int. Ed.* **52**, 4406–4409 (2013).
47. Sims, J. J., Hamou, C. A. O., Réocreux, R., Michel, C. & Giorgi, J. B. Adsorption and decomposition of formic acid on cobalt(0001). *J. Phys. Chem. C* **122**, 20279–20288 (2018).
48. Qin, Y. L., Liu, Y. C. Liang, F. & Wang, L. M. Preparation of Pd–Co-based nanocatalysts and their superior applications in formic acid decomposition and methanol oxidation.

ChemSusChem **8**, 260–263 (2015).

49. Larmier, K., Liao, W. C., Tada, S., Lam, E., Verel, R., Bansode, A., Urakawa, A., Comas-Vives, A. & Copéret, C. CO_2-to-methanol hydrogenation on zirconia-supported copper nanoparticles: Reaction intermediates and the role of the metal–support interface. *Angew. Chem. Int. Ed.* **56**, 2318–2323 (2017).
50. Tada, S., Kayamori, S., Honma, T., Kamei, H., Nariyuki, A., Kon, K., Toyao, T., Shimizu, K. & Statokawa, S. Design of interfacial sites between Cu and amorphous ZrO_2 dedicated to CO_2-to-methanol hydrogenation. *ACS Catal.* **8**, 7809−7819 (2018).
51. Hu, B., Yin, Y., Liu, G., Chen, S., Hong, X. & Tsang, S. C. E. Hydrogen spillover enabled active Cu sites for methanol synthesis from CO_2 hydrogenation over Pd doped CuZn catalysts. *J. Catal.* **359**, 17–26 (2018).
52. Zander, S., Kunkes, E. L., Schuster, M. E., Schumann, J., Weinberg, G., Teschner, D., Jacobsen, N., Schlögl, R. & Behrens, M. The role of the oxide component in the development of copper composite catalysts for methanol synthesis. *Angew. Chem. Int. Ed.* **52**, 6536–6540 (2013).
53. Nie, X., Jiang, X., Wang, H., Luo, W., Janik, M. J., Chen, Y., Guo, X. & Song, C. Mechanistic understanding of alloy effect and water promotion for Pd–Cu bimetallic catalysts in CO_2 hydrogenation to methanol. *ACS Catal.* **8**, 4873−4892 (2018).
54. Wang, Z. Q., Xu, Z. N., Peng, S. Y., Zhang, M. J., Lu, G., Chen, Q. S., Chen, Y. & Guo, G. C. High-performance and long-lived Cu/SiO_2 nanocatalyst for CO_2 hydrogenation. *ACS Catal.* **5**, 4255−4259 (2015).
55. Li, M. M. J., Chen, C., Ayvalı, T., Suo, H., Zheng, J., Teixeira, I. F., Ye, L., Zou, H, O'Hare, D. & Tsang, S. C. E. CO_2 hydrogenation to methanol over catalysts derived from single cationic layer CuZnGa LDH precursors. *ACS Catal.* **8**, 4390−4401 (2018).
56. Studt, F., Sharafutdinov, I., Abild-Pedersen, F., Elkjær, C. F., Hummelshøj, J. S., Dahl, S., Chorkendorff, I. & Nørskov, J. K. Discovery of a Ni–Ga catalyst for carbon dioxide reduction to methanol. *Nat. Chem.* **6**, 320–324 (2014).
57. Martin, O., Martín, A. J., Mondelli, C., Mitchell, S., Segawa, T. F., Hauert, R., Drouilly, C., Curulla-Ferré, D. & Pérez-Ramírez, J. Indium oxide as a superior catalyst for methanol synthesis by CO_2 hydrogenation. *Angew. Chem. Int. Ed.* **55**, 6261–6265 (2016).
58. Grabowski, R., Słoczyński, J., Śliwa, M., Mucha, D. & Socha, R. P. Influence of polymorphic ZrO_2 phases and the silver electronic state on the activity of Ag/ZrO_2 catalysts in the hydrogenation of CO_2 to methanol. *ACS Catal.* **1**, 266–278 (2011).
59. Natesakhawat, S., Lekse, J. W., Baltrus, J. P., Ohodnicki, P. R., Howard, B. H., Deng, X. & Matranga, C. Active sites and structure–activity relationships of copper-based catalysts for carbon dioxide hydrogenation to methanol. *ACS Catal.* **2**, 1667−1676 (2012).
60. Garcia-Trenco, A., White, E. R., Regoutz, A., Payne, D. J., Shaffer, M. S. P. & Williams, C. K. Pd_2Ga-based colloids as highly active catalysts for the hydrogenation of CO_2 to methanol. *ACS Catal.* **7**, 1186−1196 (2017).
61. Lunkenbein, T., Girgsdies, F., Kandemir, T., Thomas, N., Behrens, M., Schlögl, R. & Frei, E. Bridging the time gap: A copper/zinc oxide/aluminum oxide catalyst for methanol synthesis studied under industrially relevant conditions and time scales. *Angew. Chem. Int. Ed.* **55**, 12708–12712 (2016).
62. Richard, A. R. & Fan, M. Low-pressure hydrogenation of CO_2 to CH_3OH using Ni–In–Al/SiO_2 catalyst synthesized via a phyllosilicate precursor. *ACS Catal.* **7**, 5679−5692 (2017).

63. Lam, E., Larmier, K., Wolf, P., Tada, S., Safonova, O. V. & Copéret, C. Isolated Zr surface sites on silica promote hydrogenation of CO_2 to CH_3OH in supported Cu catalysts. *J. Am. Chem. Soc.* **140**, 10530−10535 (2018).
64. Bahruji, H., Bowker, M., Hutchings, G., Dimitratos, N., Wells, P., Gibson, E., Jones, W., Brookes, C., Morgan, D. & Lalev, G. Pd/ZnO catalysts for direct CO_2 hydrogenation to methanol. *J. Catal.* **343**, 133–146 (2016).
65. Chen, Y., Li, H., Zhao, W., Zhang, W., Li, J., Li, W., Zheng, X., Yan, W., Zhang, W., Zhu, J., Si, R. & Zeng, J. Optimizing reaction paths for methanol synthesis from CO_2 hydrogenation via metal-ligand cooperativity. *Nat. Commun.* **10**, 1885 (2019).
66. Li, H., Wang, L., Dai, Y., Pu, Z., Lao, Z., Chen, Y., Wang, M., Zheng, X., Zhu, J., Zhang, W., Si, R. Ma, C. & Zeng, J. Synergetic interaction between neighbouring platinum monomers in CO_2 hydrogenation *Nat. Nanotech.* **13**, 411–417 (2018).
